# Supplementary material for: Stereoselective Pharmacokinetics of Ketamine Administered at a Low Dose in Awake Dogs
Source: Animals (Basel). 2024 Mar 27;14(7):1012. doi: 10.3390/ani14071012 (PMC11011119; doi:10.3390/ani14071012)

Supplementary material S1. **Development of the pharmacokinetic models** (Monolix®)

The main steps of the model selection workflow are presented here with the results. Several intermediate steps and additional model calculations, which did not lead to model improvement, are not presented here to reduce the amount of information presented. Also, Plots of concentration time course were observed as well as residual distributions but are not fully reported here.

In this appendix, the original parameters (names) are presented as generated by Monolix®. To compare to the article, use the following equivalence:

Name in the Appendix	Name in the article / Tables
V1	$V_{(S/R)\text{-}1}$
Cl	$Cl_{(S/R)e}$
Q	$Cl_{(S/R)\text{-}12}$
V2	$V_{(S/R)\text{-}2}$
Clm	$Cl_{(S/R)\text{-}1N}$
Kpm	$Cl_{(S/R)Ne}$

Index

1. Modelling for S-Ketamine: 2
   1.1. Noncompartmental analysis (NCA): 2
   1.2. Individual compartmental analysis: 3
   1.3. Population compartmental analysis 6
2. Modelling for S-Ketamine and S-Norketamine: 12
   2.1. Individual compartmental analysis: 12
   2.2. Population compartmental analysis: 13
3. Modelling for R-Ketamine: 19
   3.1. Noncompartmental analysis (NCA): 19
   3.2. Individual compartmental analysis: 20
   3.3. Population compartmental analysis 23
4. Modelling for R-Ketamine and R-Norketamine: 30
   4.1. Individual compartmental analysis: 30
   4.2. Population compartmental analysis: 31

1. Modelling for S-Ketamine:

1.1. Noncompartmental analysis (NCA):

Model applied: Intravascular administration, “linear up log down” method for integral of AUC calculation, 3 last points for lambda_z, BLQ=LOQ/2.

Table S1_1.1.1 Median/Mean adjusted R^2 with different weighting.

Weighting	Uniform	1/Y	$1/Y^2$
Median adjusted R^2	0.91/0.87	0.91/0.88	0.94/0.89

Conclusion: The weighting $1/Y^2$ was retained.

Table S1_1.1.2 Final NCA estimates for S-Ketamine.

	Q1	median	Q3	mean	SD	SE	CV (%)	GeoMean
C_{max} (mg·L^{-1})	0.15	0.16	0.25	0.21	0.12	0.042	55.3	0.19
V_d (L·kg^{-1})	3.25	5.19	6.33	5.35	3.13	1.11	58.5	4.57
CL (L·min^{-1}·kg^{-1})	0.21	0.25	0.28	0.26	0.082	0.029	31.25	0.25
$T_{1/2}$ (min)	9.09	11.15	17.87	14.45	8.84	3.13	61.17	12.59
k_{el} (min^{-1})	0.039	0.063	0.077	0.062	0.03	0.011	49.06	0.055
$AUC_{0\text{-}inf}$ (min·mg·L^{-1})	1.79	2.01	2.36	2.05	0.55	0.19	26.68	1.99
$AUMC_{0\text{-}inf}$ (min^2·mg·L^{-1})	23.78	31.44	46.29	36.88	20.76	7.34	56.28	32.34
$MRT_{0\text{-}inf}$ (min)	10.19	14.61	21.67	17.3	10.01	3.54	57.84	15.16

1.2. Individual compartmental analysis:

Model applied: Intravenous infusion administration, no delay, mammillary 1-to-3 compartments, linear elimination, BLQ=LOQ/2.

First run with **Optimization Cost function**: $\mathbf{(Y_{pred}-Y_{obs})^2 / Y_{pred}^2}$

Table S1_1.2.1 Diagnostic values with different compartment models.

Model	1-Comp	2-Comp	3-Comp
-2LL (OFV)	-521	-608	-556
AIC	-455	-478	-362
BIC	-464	-498	-394

Figure S1_1.2.1 Observed vs. predicted concentrations with different compartment models

S1_1.2.1.a One-compartment model

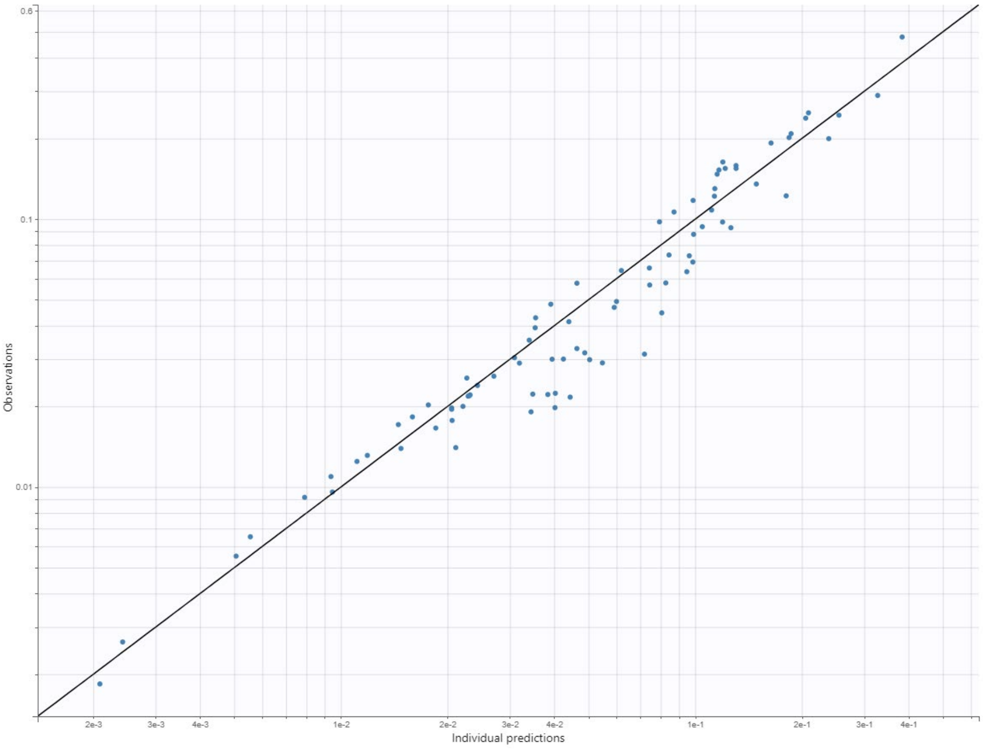

S1_1.2.1.b Two-compartments model

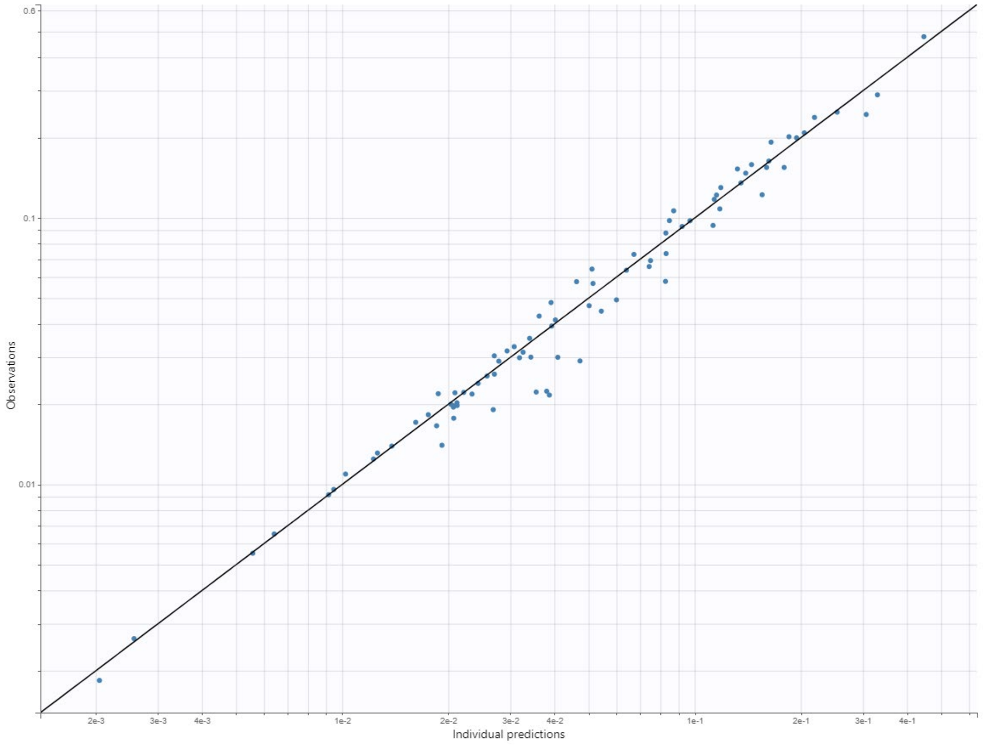

S1_1.2.1.c Three-compartments model

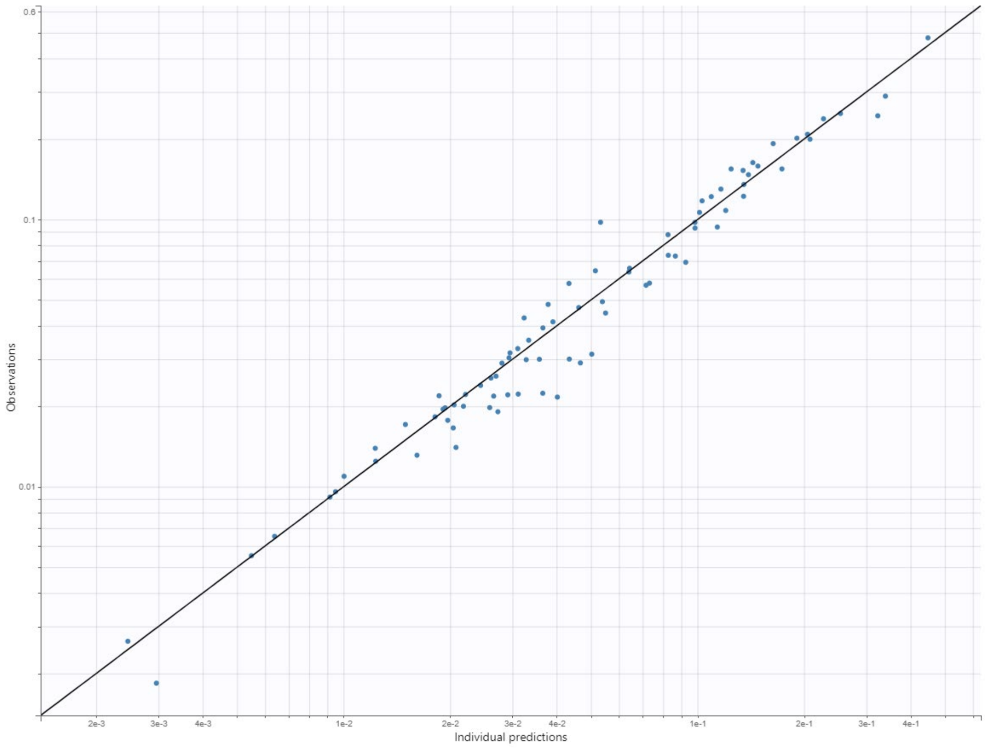

Conclusion: The two-compartments model was retained.

Definition: $Y = (Y_{pred} - Y_{obs})$

Table S1_1.2.2 Diagnostic values with different cost function used for approximation.

Cost function	Y^2	Y^2 / Y_{obs}	Y^2 / Y_{pred}	Y^2 / Y_{obs}^2	Y^2 / Y_{pred}^2	$Y^2 / \lvert Y_{pred} . Y_{obs} \rvert$
Cost	0.01	0.1	0.08	1.8	1.76	1.85
-2LL (OFV)	-562	-594	-604	-610	-606	-608
AIC	-432	-464	-474	-480	-476	-478
BIC	-452	-484	-494	-500	-496	-498

Conclusion: The Cost function $\mathbf{Y^2 / Y_{obs}^2}$ is retained.

Table S1_1.2.3 Confirmation of the most appropriate compartment model.

Model	1-Comp	2-Comp	3-Comp
-2LL (OFV)	-526	-610	-600
AIC	-460	-480	-405
BIC	-468	-500	-437

Conclusion: The Two-compartmental model is further retained.

Figure S1_1.2.2 Observed vs. predicted concentrations.

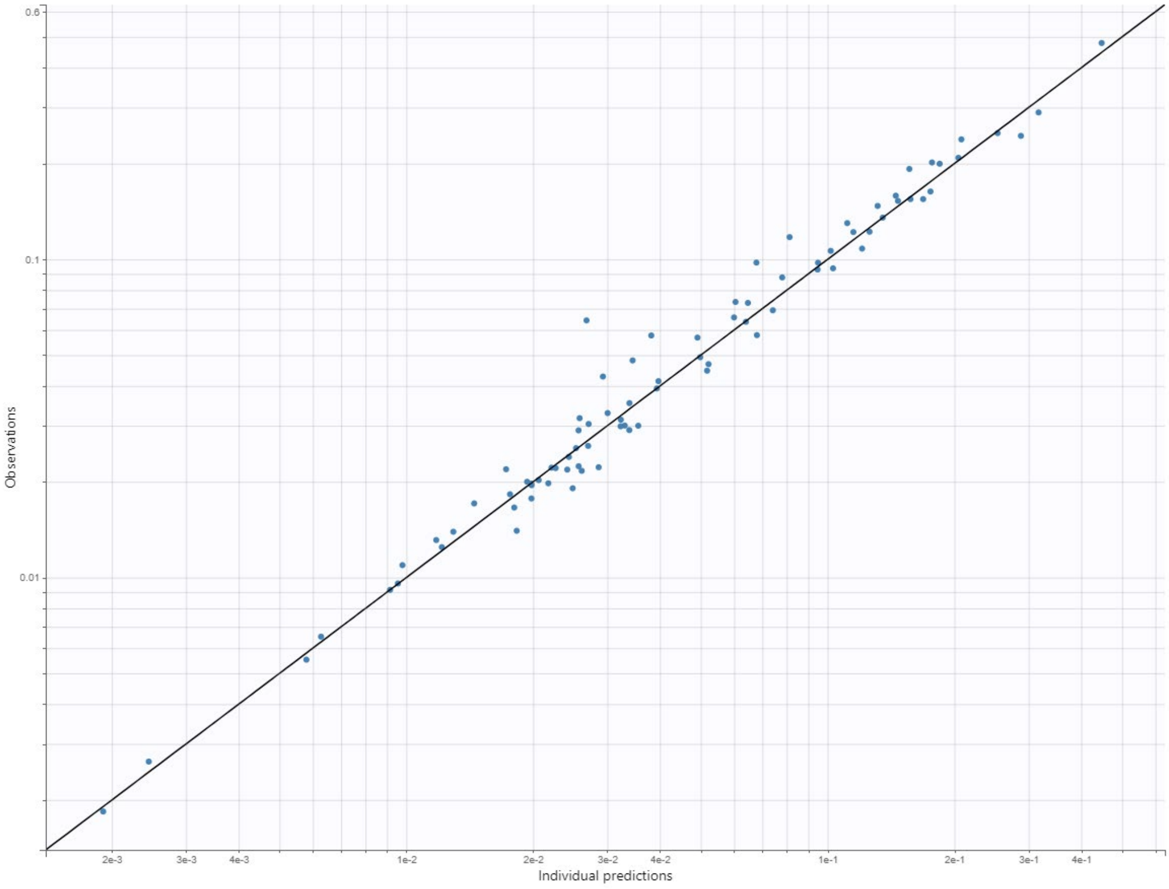

Table S1_1.2.4 Final estimates of the two-compartmental model for S-Ketamine.

	Q1	median	Q3	mean	SD	SE	CV (%)	GeoMean
Cl ($L \cdot min^{-1} \cdot kg^{-1}$)	0.16	0.2	0.24	0.21	0.065	0.016	31.59	0.2
V1 ($L \cdot kg^{-1}$)	0.55	1.27	1.65	1.3	1.02	0.25	78.09	0.93
Q ($L \cdot min^{-1} \cdot kg^{-1}$)	0.1	0.3	0.48	0.38	0.34	0.084	87.72	0.24
V2 ($L \cdot kg^{-1}$)	0.98	2.59	4.45	3.62	3.47	0.87	96.04	2.05

1.3. Population compartmental analysis

Model applied: Intravenous infusion administration, no delay, mammillary 2-compartments model, linear elimination, BLQ=LOQ/2. Concentrations are modeled with a lognormal distribution. Parameters are modeled by Stochastic Approximation Expectation-Maximization (SAEM) algorithm (Monolix©).

Main settings: Burn-in phase with 50 iterations. Exploratory phase with 250-5000 iterations (auto-stop criteria), stepsize exponent=0, enabled simulated annealing with a decreasing rate of 0.95. Smoothing phase with 100-1000 iterations (auto-stop criteria), stepsize exponent=0.7. Conditional mode with maximal 1000 iterations, tolerance at 0.000001. Stochastic approximation with 100-500 iterations. Monte Carlo size of 10000, with optimized degrees of freedom (1 to 15).
Initial estimates are adjusted, and a final run is repeated using the last final estimates.

Table S1_1.3.1 Diagnostic results with a *combined_1* error model on predicted concentration (Cp = Cc + (a + b.Cc).e), and a random effect for inter-individual variability (IIV).

Cl	V1	Q	V2	-2LL (OFV)	AIC	BIC
X				-413	-399	-394
X	X			-433	-417	-411
X		X		-437	-421	-415
X			X	-425	-409	-403
X	X	X		-437	-419	-412
X		X	X	-437	-419	-412

Conclusion: An IIV random effect is included for Cl and Q.

Table S1_1.3.2 Diagnostic results with a IIV random effect on Cl and Q:

Error Model		-2LL (OFV)	AIC	BIC
Constant	Cc + a . e	-434	-420	-415
Proportional	Cc + b . Cc . e	-431	-417	-411
Combined_1	Cc + (a + b . Cc) . e	-437	-421	-415
Combined_2	Cc + $\sqrt{(a^2 + (b \cdot cC)^2}$. e	-438	-422	-416

Table S1_1.3.3 Diagnostic results with a covariate effect (Body Weight of the individuals).

Criteria	No covariate	Weight
Cl	-2.8	-4.1
Q	28.5	30.4

Table S1_1.3.4 Diagnostic results with a Combined_2 error model on predicted concentration ($\overline{Cc + \sqrt{(a\ ^{2} + (b}$. $)^2$. e) and a random effect for inter-occasion variability (IOV), including IIV for Cl and Q, as well as Covariate effect of weight on Cl.

Cl	V1	Q	V2	-2LL (OFV)	AIC	BIC
				-442	-424	-417
X				-444	-424	-317
	X			-441	-421	-413
		X		-454	-434	-427
			X	-443	-423	-415
X		X		-458	-436	-428
	X	X		-446	-424	-416
		X	X	-453	-432	-423
X	X	X		-459	-435	-426
X		X	X	-459	-435	-426
X	X	X	X	-459	-433	-423

Table S1_1.3.5 Best final estimates for the 2-compartments model for S-ketamine including IIV and IOV for Cl and Q, a Weight-covariate effect on Cl, and a combined_2 error model.

	VALUE		STOCH. APPROX.	
			S.E.	R.S.E.(%)
**Fixed Effects**				
Cl_pop		0.32	0.14	44.8
beta_Cl_Weight_kg_		-0.056	0.047	84.1
V1_pop		1.3	0.12	9.09
Q_pop		0.11	0.042	37.3
V2_pop		3.64	1.02	28.0
**Standard Deviation of the Random Effects**				
	Value	C.V.(%)		
omega_Cl	0.0099	0.99	0.15	1.50e+3
omega_Q	0.21	21.14	0.76	365

	VALUE		STOCH. APPROX.	
			S.E.	R.S.E.(%)
gamma_Cl	0.23	23.81	0.057	24.2
gamma_Q	1.26	197.64	0.34	27.0
Error Model Parameters				
a		0.18	0.046	25.1
b		0.059	0.018	30.1

Due to observed correlations and several inappropriate RSE%, IIV is then removed stepwise.

Table S1_1.3.6 Diagnostic values for models including different IIV contributions.

Cl	V1	Q	V2	Weight	-2LL (OFV)	AIC	BIC	R.S.E.(%) > 50
				Cl	-458	-440	-434	β_{Cl_Weight}
					-457	-441	-435	
X				Cl	-459	-439	-431	β_{Cl_Weight} , ωCl
X					-457	-439	-432	ωCl
		X		Cl	-458	-438	-431	β_{Cl_Weight} , ωQ
		X			-457	-439	-432	ωQ

Table S1_1.3.7 Diagnostic results with a covariate effect (Body Weight of the individuals).

Criteria	No covariate	Weight
Cl	4.7	5.3
Q	53.8	56.4

In the final best model no IIV remains to explain parameters variability, only IOV for Cl and Q, including no covariate (Weight) effect.

Table S1_1.3.8 Validation of the error model for the final model.

Error Model		OFV	AIC	BIC	Comment
Constant	Cc + a . e	-454	-440	-435	
Proportional	Cc + b . Cc . e	-447	-433	-428	$b_{R.S.E.}$ < 10%
Combined_1	Cc + (a + b . Cc) . e	-457	-441	-434	$b_{R.S.E.}$ > 50%
Combined_2	Cc + $\sqrt{(a^2 + (b\,.\,cC)^2}$. e	-457	-441	-435	

TableS1_1.3.9 Shapiro Wilk tests for normal distribution of random effects for the final model.

	STATISTICS	P-VALUE
eta_Cl	0.96	6.87e-1
eta_Q	0.96	9.18e-1

TableS1_1.3.10 Shapiro Wilk tests for normal distribution of individual parameters for the final model.

	DISTRIBUTION	STATISTICS	P-VALUE
Cl	lognormal	0.96	6.87e-1
Q	lognormal	0.96	9.18e-1

TableS1_1.3.11 Shapiro Wilk tests for normal distribution of residuals for the final model.

	STATISTICS	P-VALUE
IWRES	0.98	5.43e-2
PWRES	0.99	8.57e-1
NPDE	0.99	5.73e-1

TableS1_1.3.12 Symmetry test around 0 for residuals for the final model

	STATISTICS	P-VALUE
IWRES	1.18	2.39e-1
PWRES	-0.13	8.94e-1
NPDE	0.47	6.39e-1

The model does not detect correlations between random effects, which may improve the model.

Table S1_1.3.13 Best final estimates of the pharmacokinetic parameters of S-Ketamine with the final model including no IIV, no covariate effect (weight), and IOV for Cl and Q.

	VALUE		STOCH. APPROX.	
			S.E.	R.S.E.(%)
**Fixed Effects**				
Cl_pop		0.19	0.018	9.56
V1_pop		1.32	0.12	8.80
Q_pop		0.11	0.042	37.1
V2_pop		3.9	1.16	29.8
**Standard Deviation of the Random Effects**				
	Value	C.V.(%)		
gamma_Cl	0.25	25.36	0.06	24.0
gamma_Q	1.29	206.94	0.34	26.0
**Error Model Parameters**				
a		0.18	0.045	24.7
b		0.059	0.017	28.5

Figure S1_1.3.1 Observed vs. predicted concentrations for S-Ketamine.

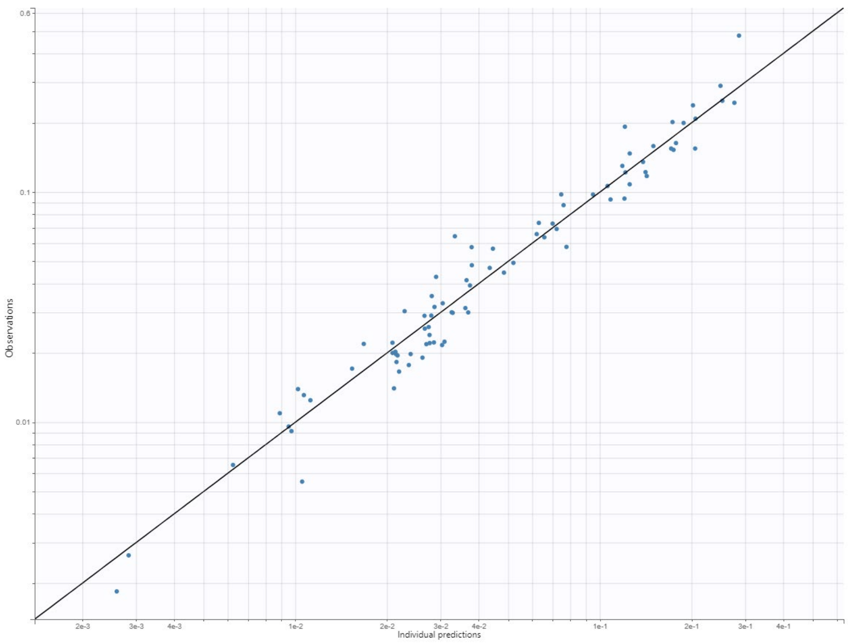

Figure S1_1.3.2 Distribution of the residuals.

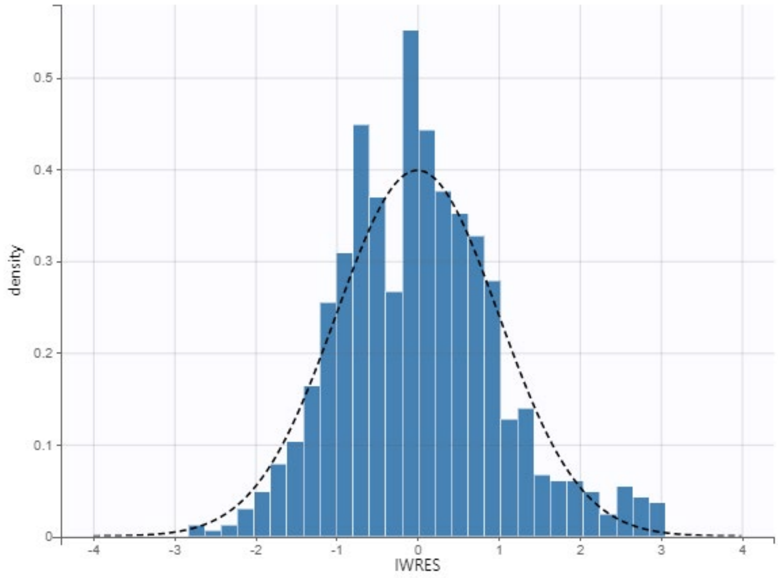

Figure S1_1.3.3 Visual predictive check of the final model.

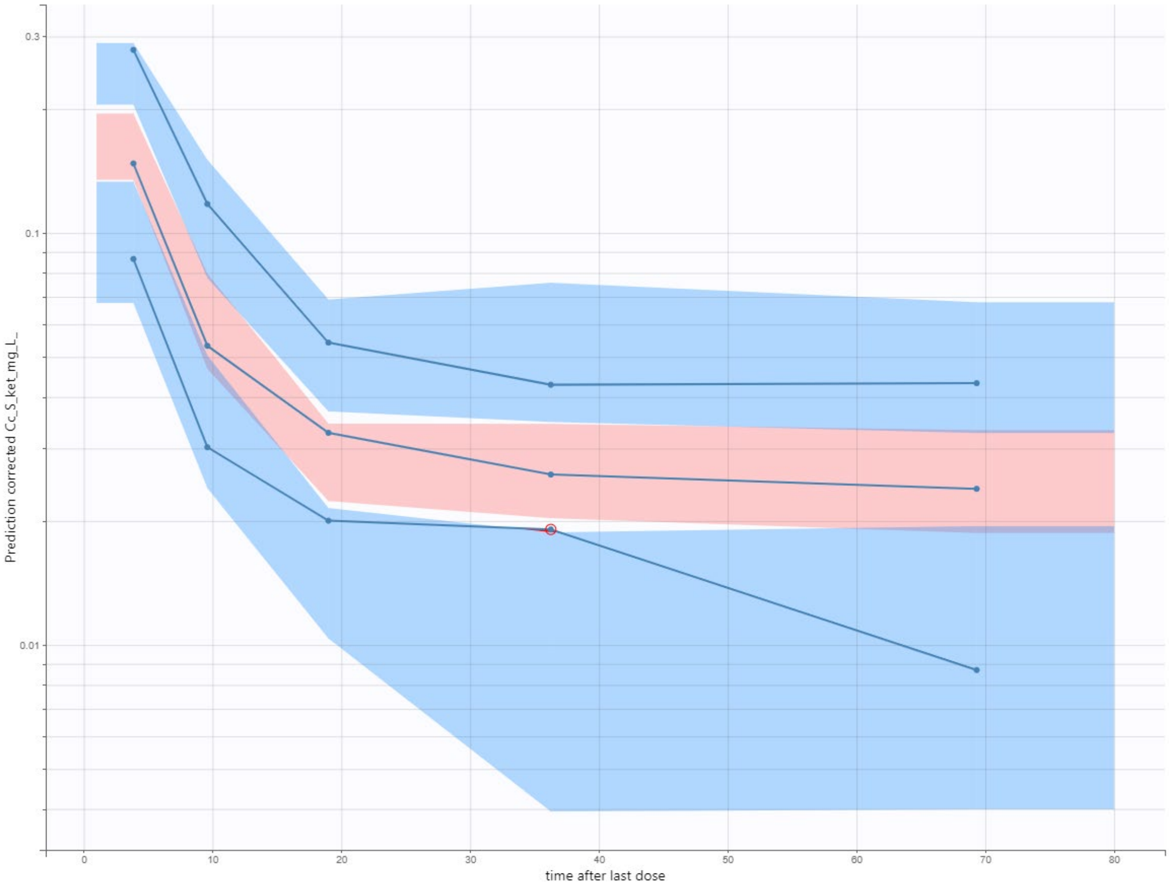

2. Modelling for S-Ketamine and S-Norketamine:

2.1. Individual compartmental analysis:

Model applied: Intravenous infusion administration, no delay, no first pass effect, unidirectional Parent-metabolite conversion, mammillary 2 compartments model with linear elimination for S-Ketamine (Parent), Linear elimination for S-Norketamine, BLQ=LOQ/2.

First run with **Optimization Cost function**: $(Y_{pred}-Y_{obs})^2 / Y_{pred}^2$

Table S1_2.1.1 Diagnostic values with different compartment models.

	Metabolite from Parent Comp 1			Metabolite from Parent Comp 2	
Model	**1-Comp**	**2-Comp**	**3-Comp**	**1-Comp**	**2-Comp**
-2LL (OFV)	-1130	-1146	-1120	-1084	-1065
AIC	-936	-888	-798	-890	-807
BIC	-900	-842	-741	-854	-761

Conclusion: The One-compartment-metabolite model issued from the first Parent-compartment was retained.

Definition: $Y = (Y_{pred}-Y_{obs})$

Table S1_2.1.2 Total cost and diagnostic values with different cost functions.

Cost function	Y^2	Y^2 / Y_{obs}	Y^2 / Y_{pred}	Y^2 / Y_{obs}^2	Y^2 / Y_{pred}^2	$Y^2 / \lvert Y_{pred} . Y_{obs} \rvert$
Cost	0.02	0.26	0.24	7.34	4.78	7.16
-2LL (OFV)	-1080	-1156	-1164	-1151	-1205	-1155
AIC	-886	-962	-970	-957	-1011	-961
BIC	-851	-926	-935	-922	-976	-925

Conclusion: The Cost function Y^2 / Y_{pred}^2 is retained.

Table S1_2.1.3 Confirmation of the best compartment model for S-Norketamine.

Model	**1-Comp**	**2-Comp**	**3-Comp**
-2LL (OFV)	-1216	-1195	-1191
AIC	-1022	-937	-869
BIC	-986	-891	-812

Conclusion: The One-compartmental model is further retained.

Figure S1_2.1.1 Observed vs. predicted concentrations with the final model (Two-compartment for S-Ketamine, One compartment issued from the central parent compartment for S-Norketamine.

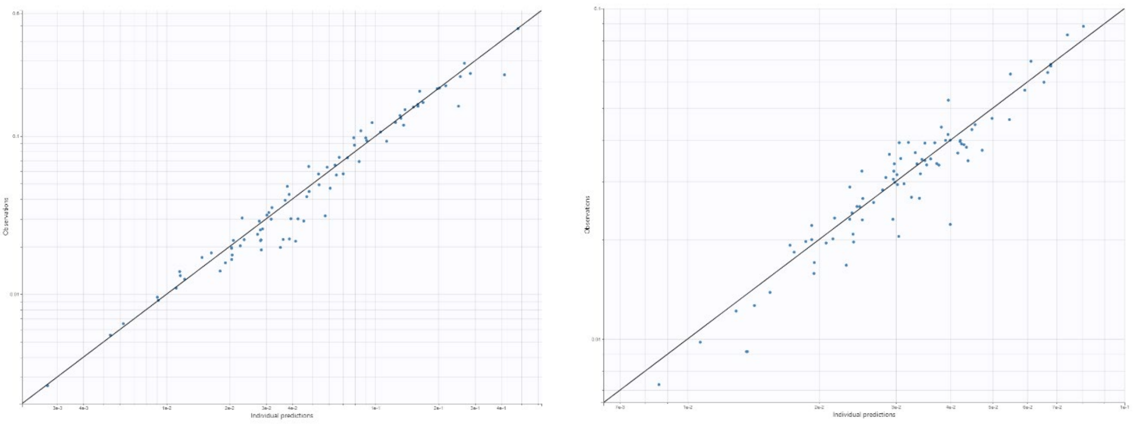

S-Ketamine (Parent) S-Norketamine (Metabolite)

Table S1_2.1.4 Best final estimates of the Parent-Metabolite model: Standard mammillary 2-Compartments model for S-Ketamine (Parent), and a standard mammillary 1-Compartment model for S-Norketamine (Metabolite), issued from the first Parent compartment.

	Q1	median	Q3	mean	SD	SE	CV (%)	GeoMean
V1	0.026	0.34	0.87	0.46	0.4	0.1	87.26	0.17
Cl	0.089	0.12	0.17	0.13	0.061	0.015	48.02	0.11
Q	0.039	0.22	0.32	0.25	0.26	0.066	107.01	0.11
V2	0.28	1.19	1.85	1.47	1.42	0.35	96.52	0.58
Clm	0.00065	0.018	0.047	0.028	0.028	0.007	98.26	0.0071
Kpm	0.014	0.041	0.069	0.045	0.034	0.0085	75.04	0.028

2.2. Population compartmental analysis:

Model applied: Intravenous infusion administration, no delay, no first pass effect, Parent-Metabolite mammillary compartments model, Parent (S-Ketamine) is modeled as a 2-compartment model, linear elimination, Metabolite (S-Norketamine)) is modeled as a 1-compartment model issued from the main parent compartment, linear elimination. BLQ=LOQ/2. Concentrations are modeled with a lognormal distribution. Parameters are modeled by Stochastic Approximation Expectation-Maximization (SAEM) algorithm (Monolix©).

Main settings: Burn-in phase with 50 iterations. Exploratory phase with 250-5000 iterations (auto-stop criteria), stepsize exponent=0, enabled simulated annealing with a decreasing rate of 0.95. Smoothing phase with 100-1000 iterations (auto-stop criteria)), stepsize exponent=0.7. Conditional mode with maximal 1000 iterations, tolerance at 0.000001. Stochastic approximation with 100-500 iterations. Monte Carlo size of 10000, with optimized degrees of freedom (1 to 15). Initial estimates are adjusted, and a final run is repeated using the last final estimates.

Table S1_2.2.1 Diagnostic results with a combined_1 error model on predicted concentration (Cp = Cc + (a + b.Cc).e) for both parent and metabolite concentrations. Based on the preliminary results, the model includes first IOV for Cl and Q for the parent model.

IIV		IOV				
Clm	Kpm	Clm	Kpm	-2LL (OFV)	AIC	BIC
				-963	-939	-930
X				-965	-939	-929
	X			-972	-946	-936
	X		X	-1000	-972	-961
	X	X		-980	-952	-942
			X	-1000	-974	-964

Conclusion: The model with IOV on Kpm is retained

Table S1_2.2.2 Diagnostic results with different error models (OFV / AIC / BIC, all values are negatives).

Parent/Metabolite	Constant	Proportional	Combined_1	Combined_2
Constant	998/976/967	1000/978/970	1000/976/967	1000/976/967
Proportional	987/965/957	989/967/958	988/964/955	989/965/955
Combined_1	998/974/965	1000/976/967	1001/975/965	1000/974/964
Combined_2	998/974/965	1001/977/967	1001/975/965	1001/975/965

Table S1_1_2.2.3 Diagnostic values to compare the use of Covariate Body weight (kg) on the model parameters (OFV / AIC / BIC, all values are negatives).

	-	Q	Kpm
-	1000/978/970		
Cl	1004/980/971	1005/979/969	1005/979/969
Q	1000/976/967		
Kpm	1000/976/967		

The model detects a **correlation between IOV random effects for Cl and Q.**

-2LL (OFV)	AIC	BIC
-1012	-986	-976

Final model equations:

log(V1) = log(V1_pop)

log(Cl) = log(Cl_pop) + β_{Cl_Weight} * Weight + γ_{Cl}

log(Q) = log(Q_pop) + γ_{Q}

log(V2) = log(V2_pop)

log(Clm) = log(Clm_pop)

log(Kpm) = log(Kpm_pop) + γ_{Kpm}

TableS1_2.2.4 Best estimates for the Parent-metabolite model for S-Ketamine and S-Norketamine with Constant and Proportional error models on S-Ketamine (Parent) and S-Norketamine (metabolite), respectively, no IIV, IOV on Cl, Q, and Kpm, and a covariate effect of Weight on Cl, and a correlation between IOV random effects for Cl and Q.

	VALUE		STOCH. APPROX. S.E.	STOCH. APPROX. R.S.E.(%)
**Fixed Effects**				
V1_pop		1.05	0.12	11.5
Cl_pop		0.42	0.23	53.7
beta_Cl_Weight_kg_		-0.13	0.061	45.8
Q_pop		0.18	0.05	27.6
V2_pop		2.82	0.36	12.9
Clm_pop		0.079	0.014	17.6
Kpm_pop		0.062	0.0078	12.6
**Standard Deviation of the Random Effects**				
	Value	C.V.(%)		
gamma_Cl	0.4	41.82	0.098	24.4
gamma_Q	0.88	108.84	0.22	24.5
gamma_Kpm	0.36	36.82	0.074	20.6
**Correlations**				
corr2_Q_Cl		0.77	0.16	21.2
**Error Model Parameters**				
a1		0.26	0.025	9.64
b2		0.066	0.006	9.17

Figure S1_2.2.1 Observed versus predicted concentrations of S-Ketamine.

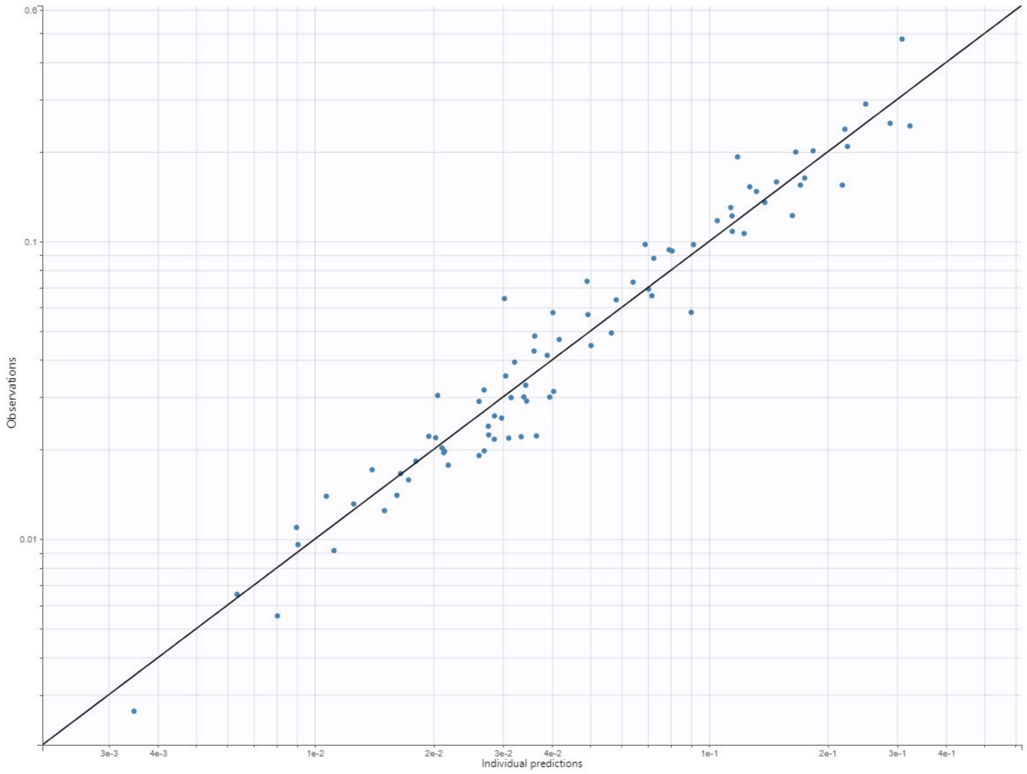

Figure S1_2.2.2 Residual distribution for S-Ketamine.

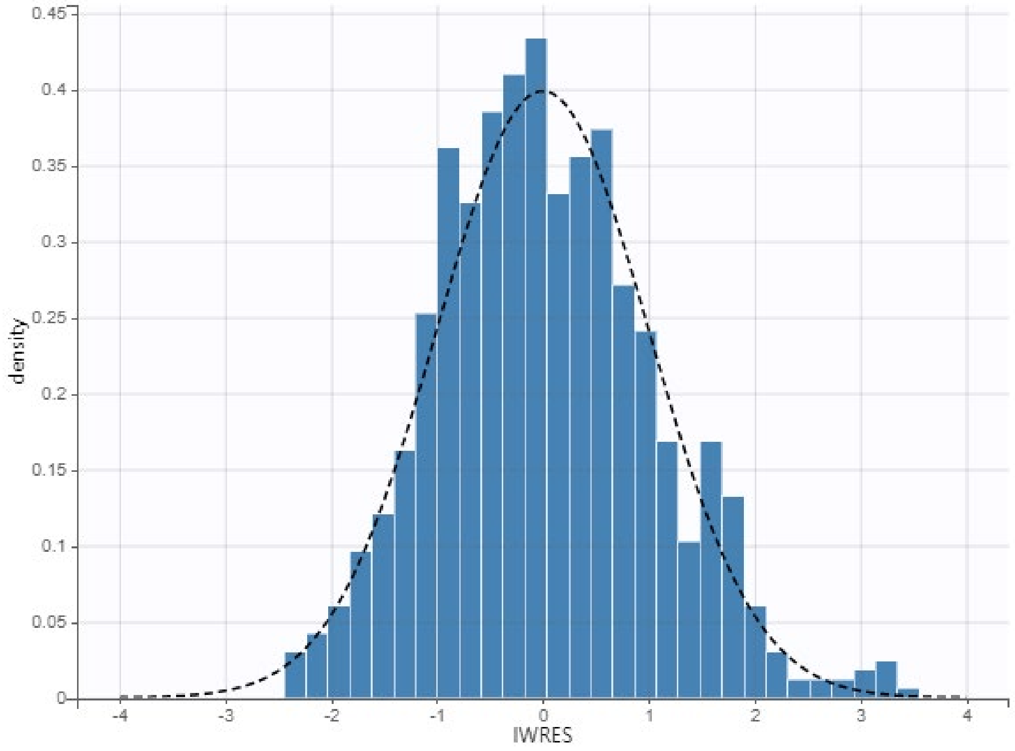

Figure S1_2.2.3 Visual predictive check for S-Ketamine.

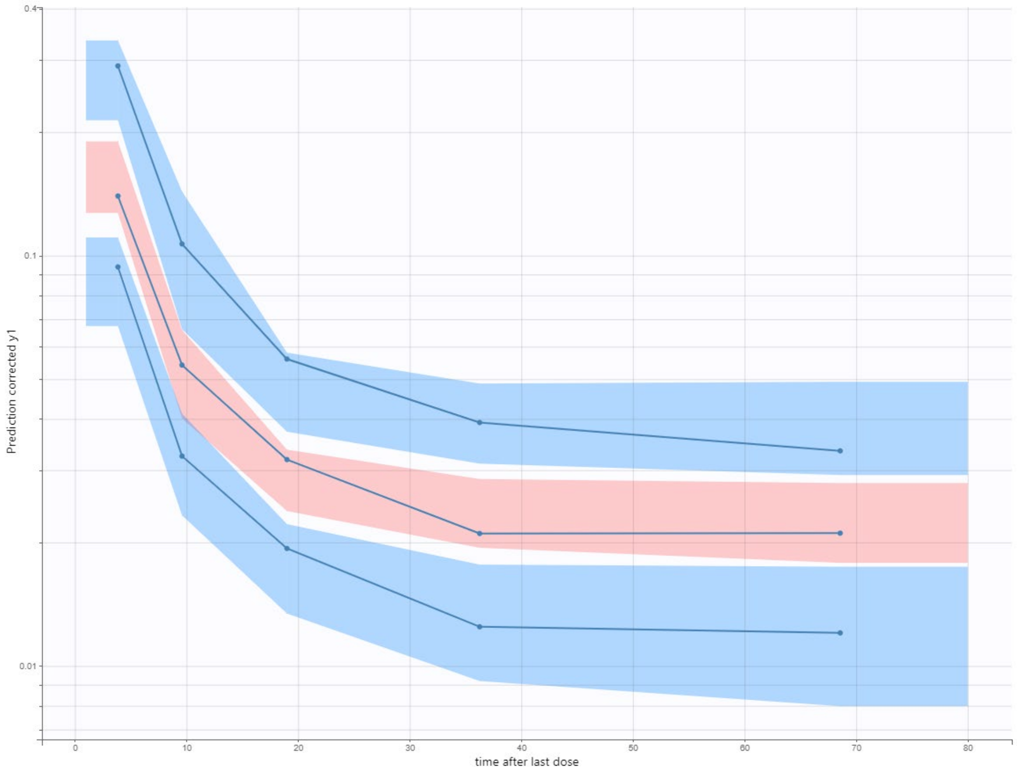

Figure S1_2.2.4 Observed versus predicted concentrations of S-Norketamine.

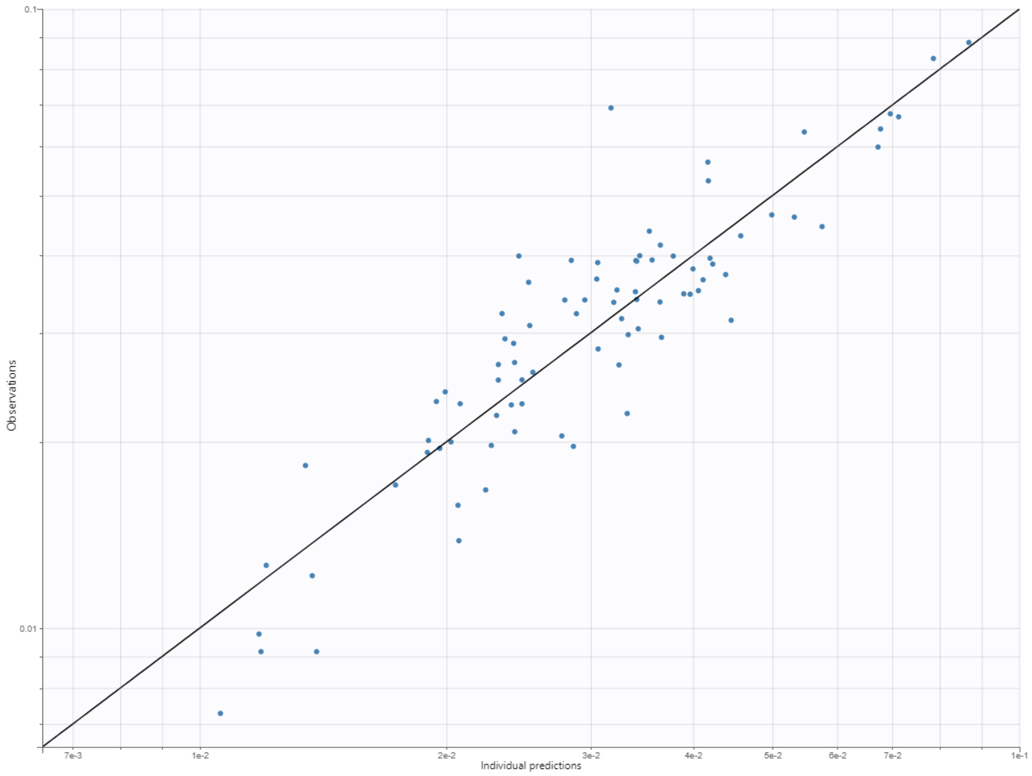

Figure S1_2.2.5 Residual distribution for S-Norketamine

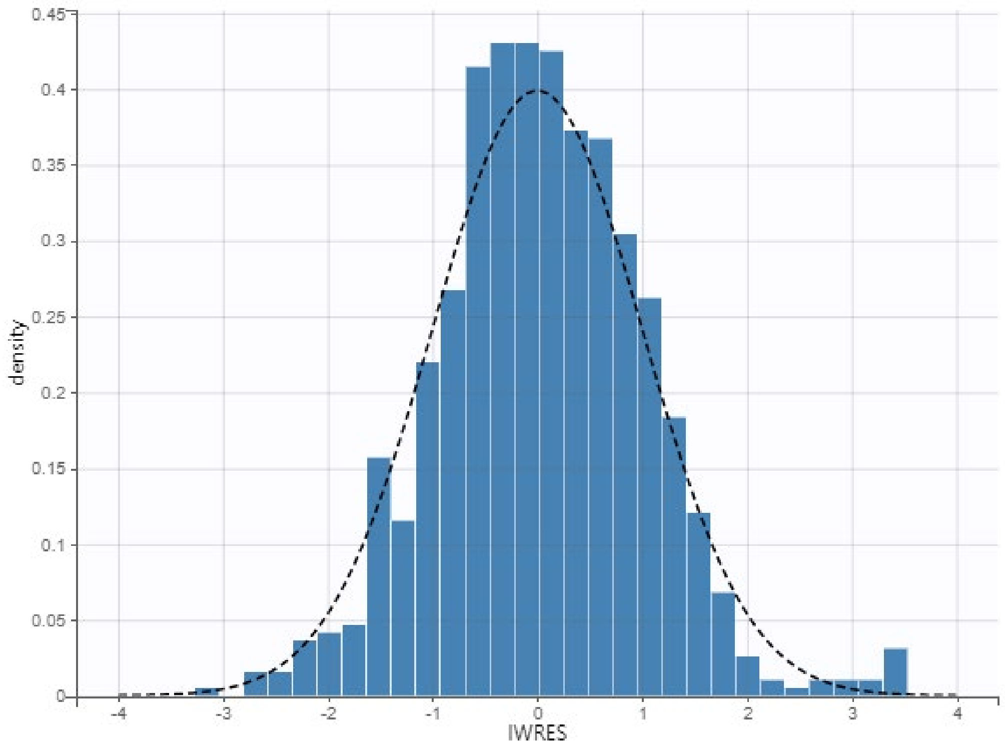

Figure S1_2.2.6 Visual predictive check for S-Norketamine.

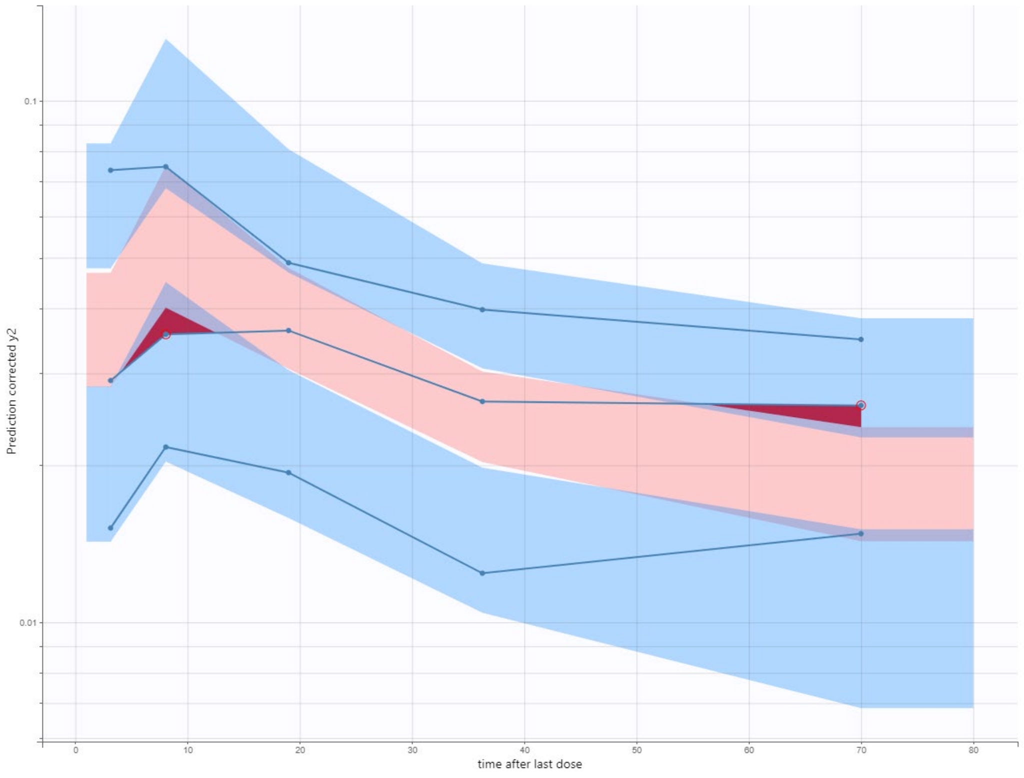

3. Modelling for R-Ketamine:

3.1. Noncompartmental analysis (NCA):

Model applied: Intravascular administration, “linear up log down” method for integral of AUC calculation, 3 last points for lambda_z, BLQ=LOQ/2.

Table S1_3.1.1 Mean and Median adjusted R^2 with different weighting.

Weighting	Uniform	1/Y	$1/Y^2$
Median adjusted R^2	0.89 ; 0.86	0.9 ; 0.86	0.93 ; 0.86

Conclusion: The weighting $1/Y^2$ was retained.

Table S1_3.1.2 Final NCA estimates for R-Ketamine.

	Q1	median	Q3	mean	SD	SE	CV (%)	GeoMean
C_{max} (mg·L^{-1})	0.14	0.15	0.22	0.19	0.096	0.034	50.4	0.17
V_d (L·kg^{-1})	3.6	6.23	7.23	6.17	3.62	1.28	58.74	5.25
CL (L·min^{-1}·kg^{-1})	0.22	0.24	0.27	0.26	0.078	0.028	29.91	0.25
$T_{1/2}$ (min)	10.99	13.6	19.82	16.44	9.5	3.36	57.76	14.42
k_{el} (min^{-1})	0.035	0.052	0.063	0.054	0.03	0.01	54.41	0.048
$AUC_{0\text{-}inf}$ (min·mg·L^{-1})	1.89	2.07	2.28	2.03	0.45	0.16	22.05	1.98
$AUMC_{0\text{-}inf}$ (min^2·mg·L^{-1})	24.32	38.09	53.76	41.58	22.13	7.82	53.21	36.76
$MRT_{0\text{-}inf}$ (min)	11.9	17.92	24.38	19.81	11.04	3.9	55.73	17.43

3.2. Individual compartmental analysis:

Model applied: Intravenous infusion administration, no delay, mammillary 1-to-3 compartments, linear elimination, BLQ=LOQ/2.

First run with **Optimization Cost function**: **$(Y_{pred}-Y_{obs})^2$ / Y_{pred}^2**

Table S1_3.2.1 Diagnostic values with different compartment models.

Model	1-Comp	2-Comp	3-Comp
-2LL (OFV)	-527	-562	-548
AIC	-461	-432	-354
BIC	-470	-453	-387

Figure S1_3.2.1 Observed vs. predicted concentrations with different compartment models

S1_3.2.1.a One-compartment model

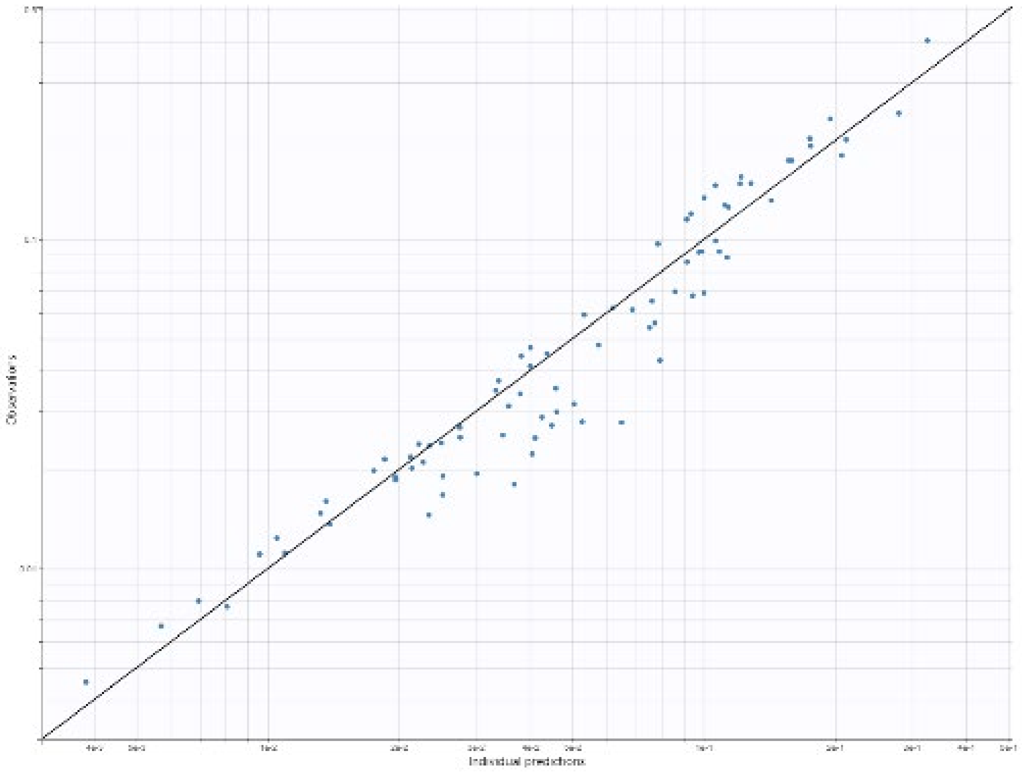

S1_3.2.1.a Two-compartment model

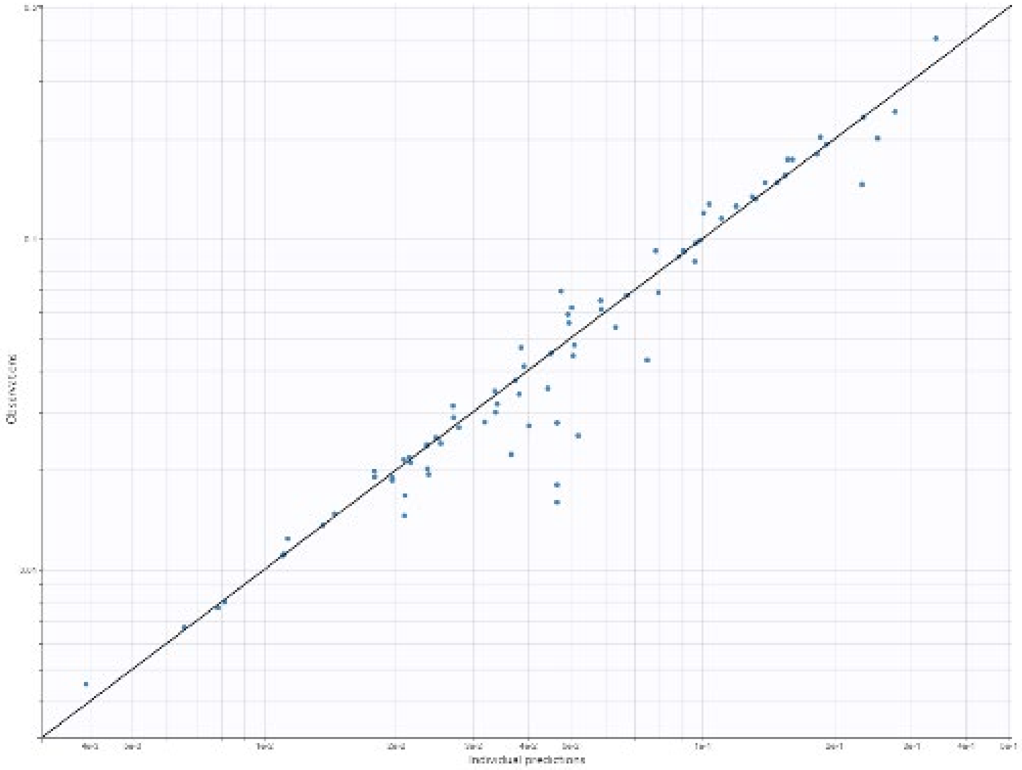

S1_3.2.1.a Three-compartment model

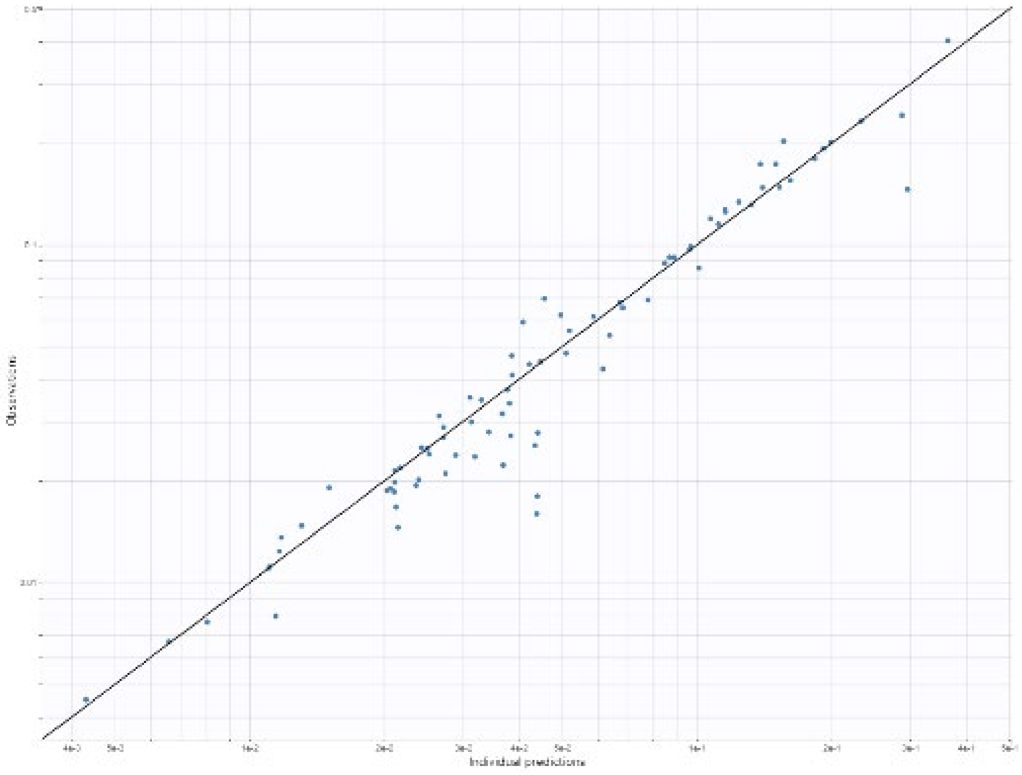

Conclusion: The two-compartment model was retained.

Definition: $Y = (Y_{pred}-Y_{obs})$

Table S1_3.2.2 Diagnostic values with different cost function used for approximation.

Cost function	Y^2	Y^2 / Y_{obs}	Y^2 / Y_{pred}	Y^2 / Y_{obs}^2	Y^2 / Y_{pred}^2	$Y^2 / \lvert Y_{pred} . Y_{obs} \rvert$
Cost	0.01	0.12	0.08	1.42	2.59	1.63
-2LL (OFV)	-539	-569	-598	-621	-562	-610
AIC	-409	-439	-468	-491	-432	-480
BIC	-430	-460	-489	-512	-453	-501

Conclusion: The Cost function $\mathbf{Y^2 / Y_{obs}^2}$ is retained.

Table S1_3.2.3 Confirmation of the most appropriate compartment model.

Model	1-Comp	2-Comp	3-Comp
-2LL (OFV)	-530	-621	-593
AIC	-464	-491	-399
BIC	-473	-512	-431

Conclusion: The Two-compartmental model is further retained.

Figure S1_3.2.2 Observed vs. predicted concentrations.

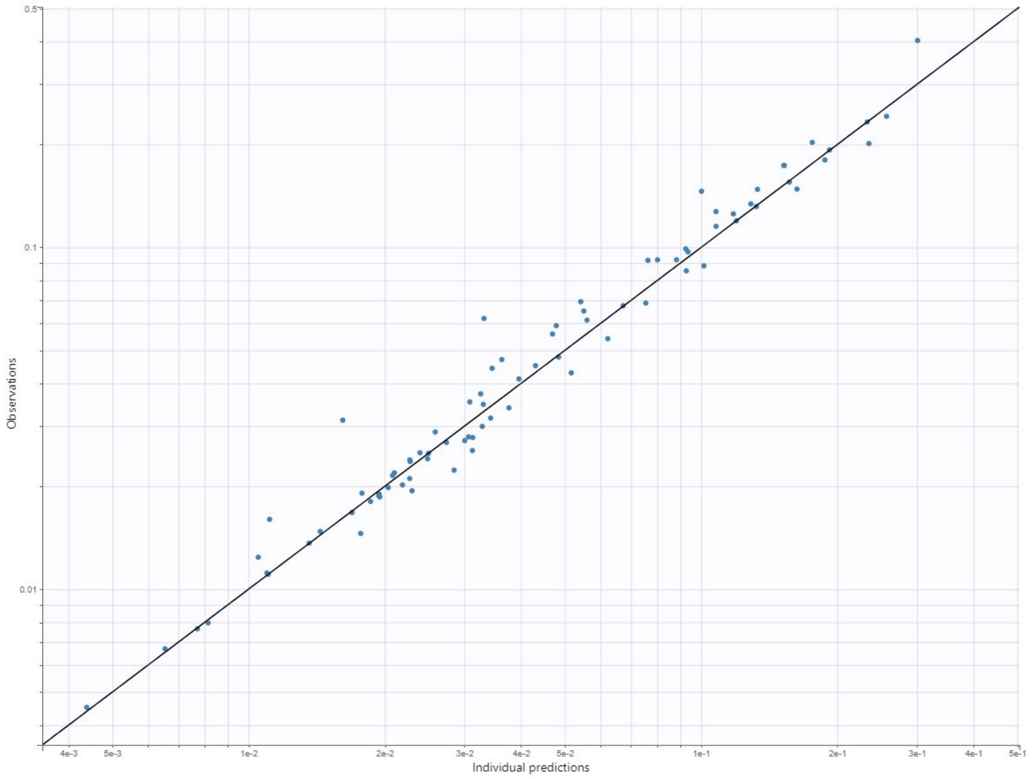

Table S1_3.2.4 Final estimates of the two-compartmental model for R-Ketamine.

	Q1	median	Q3	mean	SD	SE	CV (%)	GeoMean
Cl ($L{\cdot}min^{-1}{\cdot}kg^{-1}$)	0.12	0.2	0.22	0.21	0.18	0.045	84.25	0.13
V1 ($L{\cdot}kg^{-1}$)	0.7	1.6	3.13	2.11	1.87	0.47	88.73	1.34
Q ($L{\cdot}min^{-1}{\cdot}kg^{-1}$)	0.24	0.35	0.76	1.13	2.44	0.61	215.99	0.42
V2 ($L{\cdot}kg^{-1}$)	3.01	5.01	31.29	19.66	27.3	6.82	138.85	4.24

3.3. Population compartmental analysis

Model applied: Intravenous infusion administration, no delay, mammillary 2-compartments model, linear elimination, BLQ=LOQ/2. Concentrations are modeled with a lognormal distribution. Parameters are modeled by Stochastic Approximation Expectation-Maximization (SAEM) algorithm (Monolix©).

Main settings: Burn-in phase with 50 iterations. Exploratory phase with 250-5000 iterations (auto-stop criteria), stepsize exponent=0, enabled simulated annealing with a decreasing rate of 0.95. Smoothing phase with 100-1000 iterations (auto-stop criteria), stepsize exponent=0.7. Conditional mode with maximal 1000 iterations, tolerance at 0.000001. Stochastic approximation with 100-500 iterations. Monte Carlo size of 10000, with optimized degrees of freedom (1 to 15).
Initial estimates are adjusted, and a final run is repeated using the last final estimates.

Table S1_3.3.1 Diagnostic results with a *combined_1* error model on predicted concentration (Cp = Cc + (a + b.Cc).e), and a random effect for inter-individual variability (IIV).

Cl	V1	Q	V2	-2LL (OFV)	AIC	BIC
X				-398	-384	-379
X	X			-416	-400	-394
X		X		-412	-396	-390
X			X	-406	-390	-384
X	X	X		-416	-398	-391
X	X		X	-415	-397	-391

Conclusion: An IIV random effect is included for Cl and V1.

Table S1_3.3.2 Diagnostic results with a IIV random effect on Cl and Q:

Error Model		-2LL (OFV)	AIC	BIC
Constant	Cc + a . e	-416	-402	-396
Proportional	Cc + b . Cc . e	-407	-393	-387
Combined_1	Cc + (a + b . Cc) . e	-416	-400	-394
Combined_2	Cc + $\sqrt{(a^2 + (b\,.\,cC)^2}$. e	-416	-400	-394

Table S1_3.3.3 Diagnostic results with a covariate effect (Body Weight of the individuals).

Criteria	No covariate	Weight
Cl	-25	-23
V1	31	32

Table S1_3.3.4 Diagnostic results with a Constant error model on predicted concentration (Cc + a . e) and a random effect for inter-occasion variability (IOV), including IIV for Cl and V1, without a Covariate effect (Body weight).

Cl	V1	Q	V2	-2LL (OFV)	AIC	BIC
				-416	-402	-396
X				-436	-420	-414
	X			-429	-413	-406
		X		-426	-410	-404
			X	-429	-413	-407
X	X			-440	-422	-415
X		X		-452	-434	-427
X			X	-437	-419	-412
X	X	X		-452	-433	-425
X		X	X	-452	-432	-424

Table S1_3.3.5 Best final estimates for the 2-compartments model for R-ketamine including IIV for Cl and V1, and IOV for Cl and Q, without covariate effect, and a constant error model.

	VALUE		STOCH. APPROX.	
			S.E.	R.S.E.(%)
**Fixed Effects**				
Cl_pop		0.2	0.024	11.6
V1_pop		1.58	0.19	11.7
Q_pop		0.19	0.065	34.4
V2_pop		4.91	0.74	15.1
**Standard Deviation of the Random Effects**				
	Value	C.V.(%)		
omega_Cl	0.011	1.15	0.15	1.35e+3
omega_V1	0.064	6.42	0.26	412
gamma_Cl	0.41	43.03	0.08	19.5
gamma_Q	1.14	164.52	0.32	27.8

	VALUE	STOCH. APPROX.	
		S.E.	R.S.E.(%)
Error Model Parameters			
a	0.23	0.023	9.83

Due to observed correlations and several inappropriate RSE%, IIV is then removed stepwise.

Table S1_3.3.6 Diagnostic values for models including different IIV contributions.

Cl	V1	Q	V2	-2LL (OFV)	AIC	BIC	R.S.E.(%) > 50
				-452	-438	-433	
X				-452	-436	-430	ωCl
	X			-452	-436	-430	ωV1
		X		-452	-436	-430	ωQ
X	X			-452	-434	-427	ωCl, ωV1

The model detects a correlation between Cl and Q for IOV:

-2LL (OFV)	AIC	BIC	R.S.E.(%) > 50
-458	-442	-436	

Table S1_3.3.7 Diagnostic results with a covariate effect (Body Weight of the individuals).

Criteria	No covariate	Weight
Cl	21.16	22.68
Q	49.11	51.86

In the final best model no IIV remains to explain parameters variability, only IOV for Cl and Q, including no covariate (Weight) effect, and a correlation between Cl and Q IOV.

Table S1_3.3.8 Validation of the error model for the final model.

Error Model		OFV	AIC	BIC	Comment
Constant	Cc + a . e	-458	-442	-436	
Proportional	Cc + b . Cc . e	-448	-432	-426	$b_{R.S.E.}$ < 10%
Combined_1	Cc + (a + b . Cc) . e	-458	-440	-433	$b_{R.S.E.}$ > 50%
Combined_2	$Cc + \sqrt{(a^2 + (b \cdot cC)^2} \cdot e$	-458	-440	-433	$a_{R.S.E}$ & $b_{R.S.E.}$ > 50%

Table S1_3.3.9 Shapiro Wilk tests for normal distribution of random effects for the final model.

	STATISTICS	P-VALUE
eta_Cl	0.95	6.89e-1
eta_Q	0.94	7.3e-1

Table S1_3.3.10 Shapiro Wilk tests for normal distribution of individual parameters for the final model.

	DISTRIBUTION	STATISTICS	P-VALUE
Cl	lognormal	0.95	6.89e-1
Q	lognormal	0.94	7.3e-1

Table S1_3.3.11 Shapiro Wilk tests for normal distribution of residuals for the final model.

	STATISTICS	P-VALUE
IWRES	0.98	3.53e-1
PWRES	0.98	2.66e-1
NPDE	0.99	6.3e-1

Table S1_3.3.12 Symmetry test around 0 for residuals for the final model

	STATISTICS	P-VALUE
IWRES	0.62	5.34e-1
PWRES	-1.14	2.56e-1
NPDE	-1.32	1.88e-1

TableS1_3.3.13 Best final estimates of the pharmacokinetic parameters of R-Ketamine with the final model including a constant error model, no IIV, no covariate effect (weight), and IOV for Cl and Q including correlation.

	VALUE		STOCH. APPROX.	
			S.E.	R.S.E.(%)
**Fixed Effects**				
Cl_pop		0.2	0.024	11.6
V1_pop		1.52	0.15	10.2
Q_pop		0.19	0.059	30.9
V2_pop		4.67	0.64	13.7
**Standard Deviation of the Random Effects**				
	Value	C.V.(%)		
gamma_Cl	0.43	44.82	0.082	19.2
gamma_Q	1.05	142.83	0.25	23.4
**Correlations**				
corr2_Q_Cl		0.64	0.19	29.9
**Error Model Parameters**				
a		0.24	0.023	9.69

Figure S1_3.3.1 Observed vs. predicted concentrations for R-Ketamine.

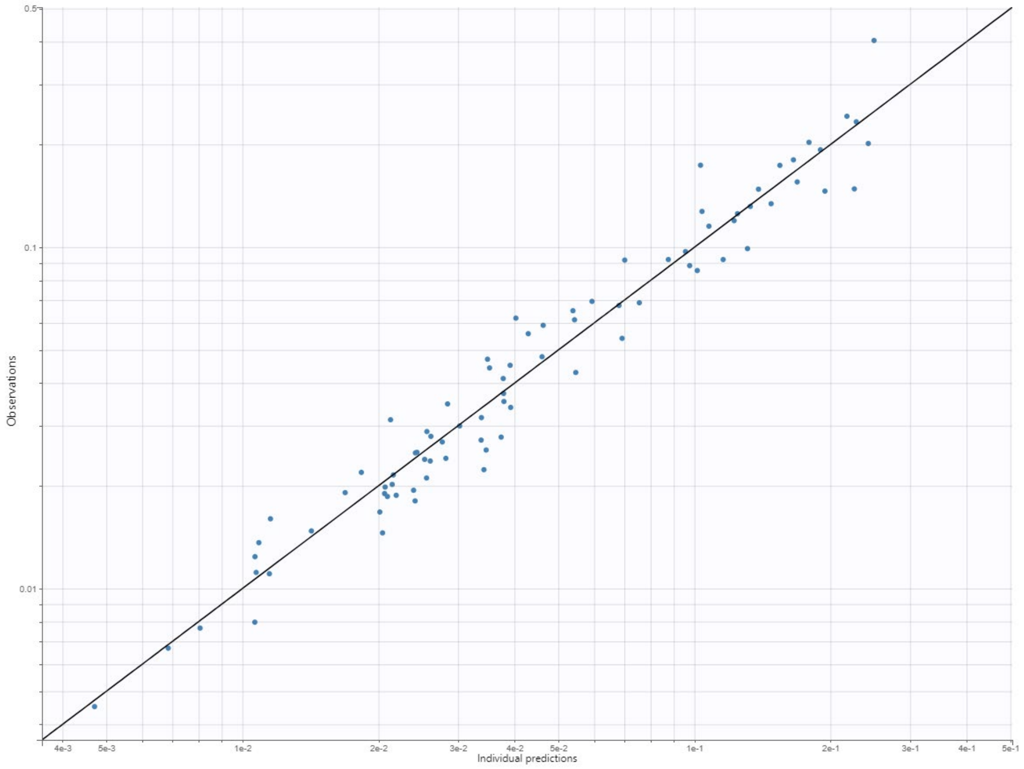

Figure S1_3.3.2 Distribution of the residuals.

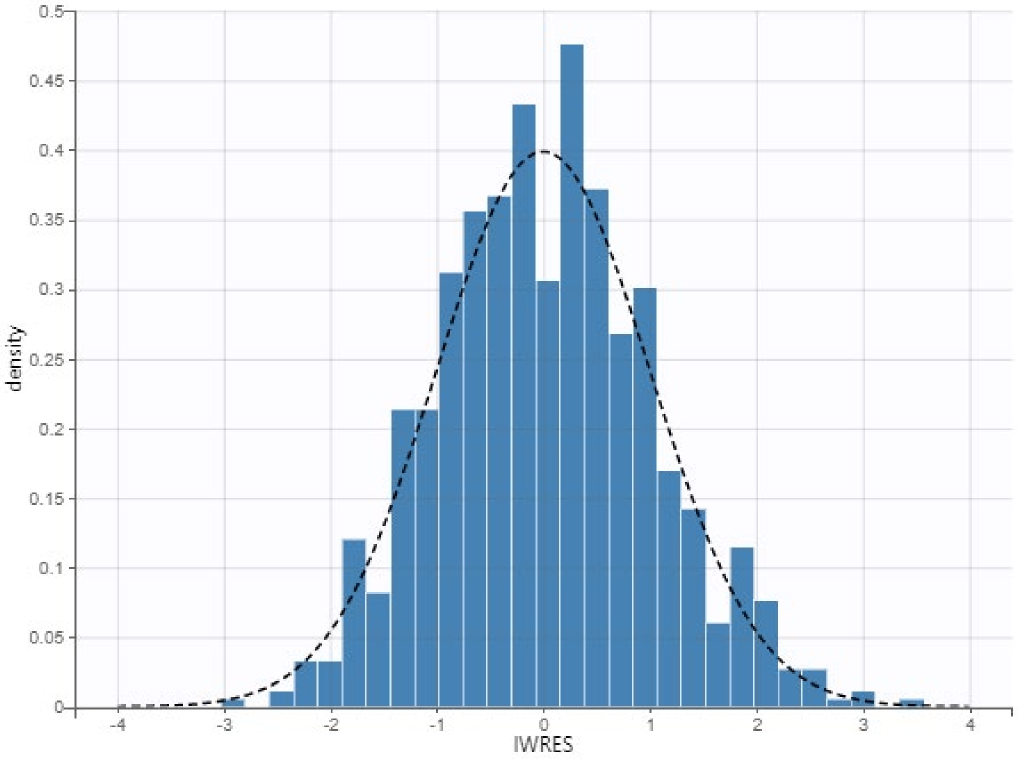

Figure S1_3.3.3 Visual predictive check of the final model.

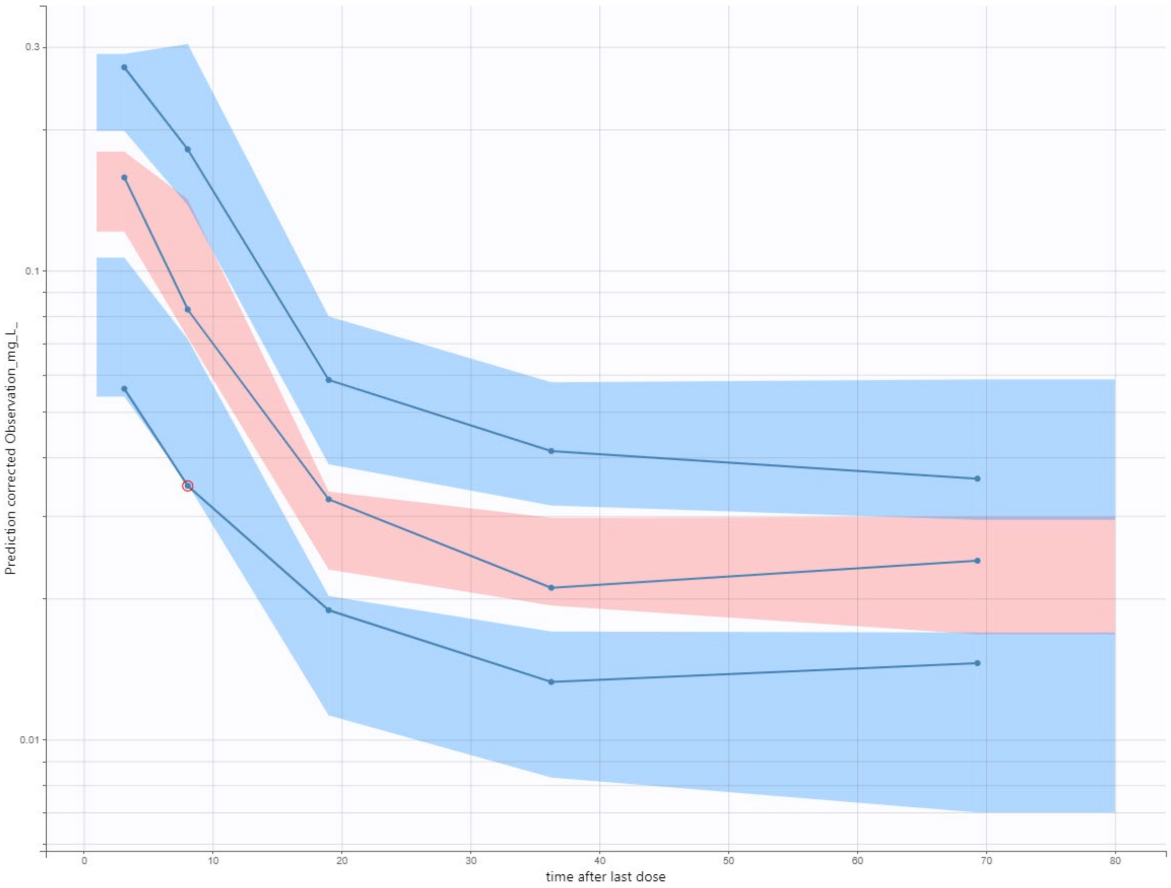

4. Modelling for R-Ketamine and R-Norketamine:

4.1. Individual compartmental analysis:

Model applied: Intravenous infusion administration, no delay, no first pass effect, unidirectional Parent-metabolite conversion, mammillary 2 compartments model with linear elimination for S-Ketamine (Parent), Linear elimination for S-Norketamine, BLQ=LOQ/2.

First run with **Optimization Cost function**: $\mathbf{(Y_{pred}-Y_{obs})^2 / Y_{pred}^2}$

Table S1_4.1.1 Diagnostic values with different compartment models.

	Metabolite from Parent Comp 1			Metabolite from Parent Comp 2	
Model	**1-Comp**	**2-Comp**	**3-Comp**	**1-Comp**	**2-Comp**
-2LL (OFV)	-1206	-1184	-1178	-1143	-1192
AIC	-1012	-926	-856	-949	-934
BIC	-977	-880	-799	-913	-888

Conclusion: The One-compartment-metabolite model issued from the first Parent-compartment was retained.

Definition: $Y = (Y_{pred}-Y_{obs})$

Table S1_4.1.2 Total cost and diagnostic values with different cost functions.

Cost function	Y^2	Y^2 / Y_{obs}	Y^2 / Y_{pred}	Y^2 / Y_{obs}^2	Y^2 / Y_{pred}^2	$Y^2 / \lvert Y_{pred} .Y_{obs} \rvert$
Cost	0.01	0.18	0.18	6.73	6.31	6.93
-2LL (OFV)	-1196	-1241	-1239	-1217	-1206	-1211
AIC	-1002	-1047	-1045	-1023	-1012	-1017
BIC	-966	-1012	-1010	-988	-977	-982

Conclusion: The Cost function $\mathbf{Y^2 / Y_{obs}}$ is retained.

Table S1_4.1.3 Confirmation of the best compartment model for S-Norketamine.

Model	**1-Comp**	**2-Comp**	**3-Comp**
-2LL (OFV)	-1261	-1200	-1160
AIC	-1067	-942	-838
BIC	-1032	-896	-781

Conclusion: The One-compartmental model is further retained.

Figure S1_4.1.1 Observed vs. predicted concentrations with the final model (Two-compartment for S-Ketamine, One compartment issued from the central parent compartment for S-Norketamine.

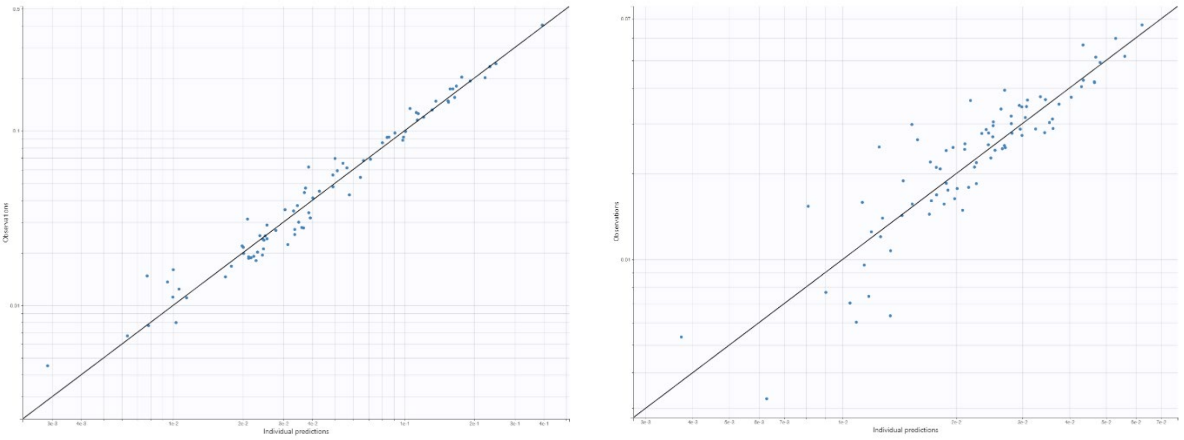

S-Ketamine (Parent) S-Norketamine (Metabolite)

TableS1_4.1.4 Best final estimates of the Parent-Metabolite model: Standard mammillary 2-Compartments model for S-Ketamine (Parent), and a standard mammillary 1-Compartment model for S-Norketamine (Metabolite), issued from the first Parent compartment.

	Q1	median	Q3	mean	SD	SE	CV (%)	GeoMean
V1	0.12	0.7	1.18	0.83	0.76	0.19	91.08	0.22
Cl	0.13	0.19	0.22	0.17	0.063	0.016	36.88	0.16
Q	0.17	0.32	0.95	0.83	1.32	0.33	158.88	0.27
V2	1.82	3	4.5	3.14	1.6	0.4	51.03	2.67
Clm	0.00092	0.04	0.1	0.074	0.11	0.028	151.23	0.012
Kpm	0.03	0.048	0.086	0.056	0.034	0.0084	60.15	0.047

4.2. Population compartmental analysis:

Model applied: Intravenous infusion administration, no delay, no first pass effect, Parent-Metabolite mammillary compartments model, Parent (S-Ketamine) is modeled as a 2-compartment model, linear elimination, Metabolite (S-Norketamine)) is modeled as a 1-compartment model issued from the main parent compartment, linear elimination. BLQ=LOQ/2. Concentrations are modeled with a lognormal distribution. Parameters are modeled by Stochastic Approximation Expectation-Maximization (SAEM) algorithm (Monolix©).

Main settings: Burn-in phase with 50 iterations. Exploratory phase with 250-5000 iterations (auto-stop criteria), stepsize exponent=0, enabled simulated annealing with a decreasing rate of 0.95. Smoothing phase with 100-1000 iterations (auto-stop criteria), stepsize exponent=0.7. Conditional mode with maximal 1000 iterations, tolerance at 0.000001. Stochastic approximation with 100-500 iterations. Monte Carlo size of 10000, with optimized degrees of freedom (1 to 15). Initial estimates are adjusted, and a final run is repeated using the last final estimates.

Table S1_4.2.1 Diagnostic results with a constant error model on predicted concentration (Cp = Cc + a.e) for both parent and metabolite concentrations. Based on the preliminary results, the model includes first IOV for Cl and Q for the parent model.

IIV		IOV				
Clm	Kpm	Clm	Kpm	-2LL (OFV)	AIC	BIC
				-993	-971	-963
X				-995	-971	-962
	X			-1004	-980	-971
		X		-997	-973	-963
			X	-1006	-982	-973
	X		X	-1007	-981	-971
		X	X	-1007	-981	-971

Conclusion: The model with IOV on Kpm is retained

Table S1_4.2.2 Diagnostic results with different error models (OFV / AIC / BIC, all values are negatives).

Parent/Metabolite	Constant	Proportional	Combined_1	Combined_2
Constant	1006/982/973	1018/994/984	1017/991/981	1017/991/981

Table S1_4.2.3 Diagnostic values to compare the use of Covariate Body weight (kg) on the model parameters (OFV / AIC / BIC, all values are negatives).

-	1006/982/973
Kpm	1020/995/985

Final model equations:

$\log(V1) = \log(V1\_pop)$

$\log(Cl) = \log(Cl\_pop) + \gamma_{Cl}$

$\log(Q) = \log(Q\_pop) + \gamma_{Q}$

$\log(V2) = \log(V2\_pop)$

$\log(Clm) = \log(Clm\_pop)$

$\log(Kpm) = \log(Kpm\_pop) + \beta_{Kpm\_Weight} * Weight + \gamma_{Kpm}$

TableS1_4.2.4 Best estimates for the Parent-metabolite model for R-Ketamine and R-Norketamine with Constant and Proportional error models on R-Ketamine (Parent) and R-Norketamine (metabolite), respectively, no IIV, IOV on Cl, Q, and Kpm, and a covariate effect of Weight on Kpm, correlation between Cl and Q.

	VALUE		STOCH. APPROX.	
			S.E.	R.S.E.(%)
**Fixed Effects**				
V1_pop	1.52		0.13	8.31
Cl_pop	0.11		0.019	16.8
Q_pop	0.16		0.043	26.4
V2_pop	4.14		0.69	16.8
Clm_pop	0.091		0.014	15.4
Kpm_pop	0.016		0.0083	50.4
beta_Kpm_Weight_kg_	0.11		0.053	49.6
**Standard Deviation of the Random Effects**				
	Value	C.V.(%)		
gamma_Cl	0.46	49.11	0.1	22.4
gamma_Q	0.92	114.92	0.21	23.3
gamma_Kpm	0.25	25.67	0.084	33.3
**Correlations**				
corr2_Q_Cl	0.61		0.21	35.0
**Error Model Parameters**				
a1	0.22		0.022	10.2
b2	0.097		0.0089	9.15

Figure S1_4.2.1 Observed versus predicted concentrations of R-Ketamine.

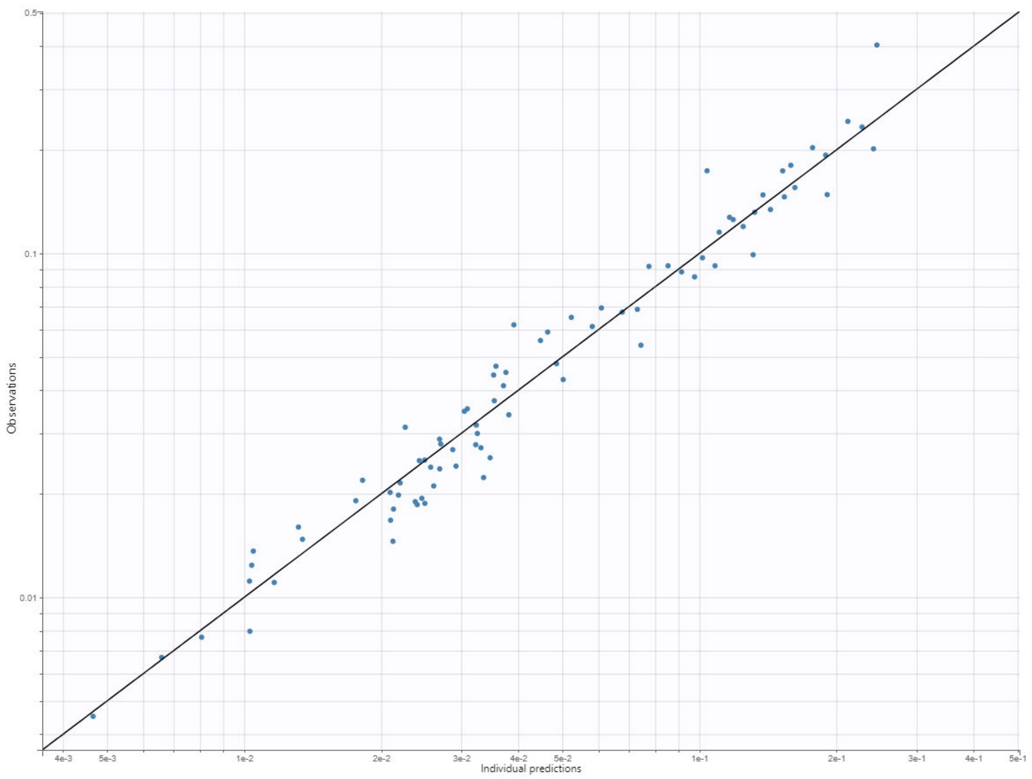

Figure S1_4.2.2 Residual distribution for R-Ketamine.

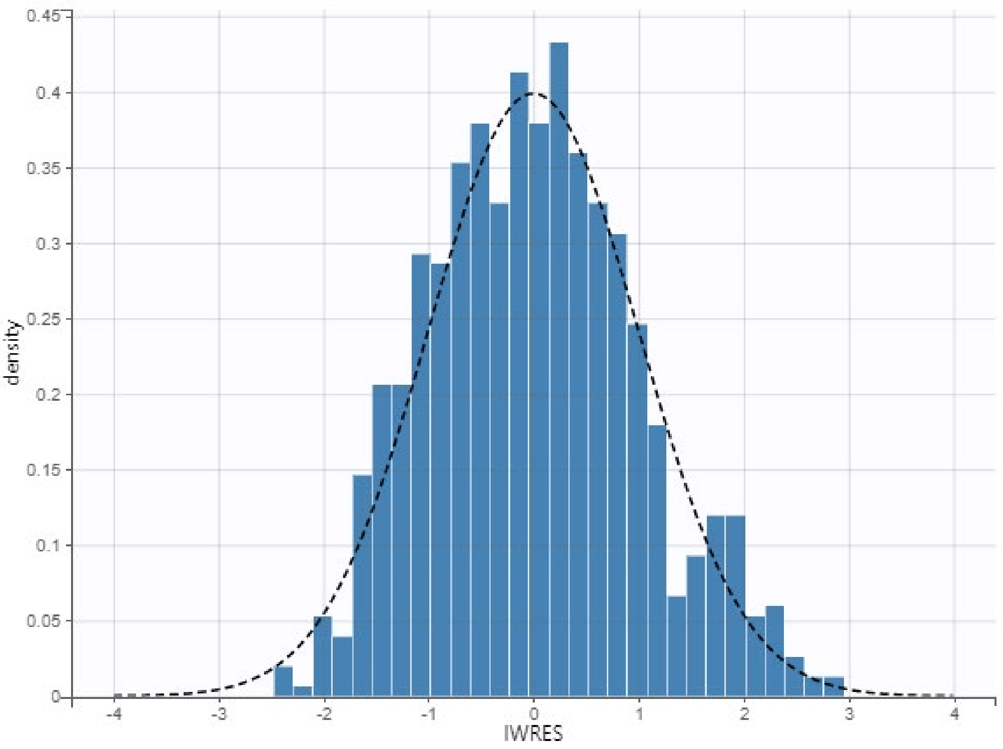

Figure S1_4.2.3 Visual predictive check for S-Ketamine.

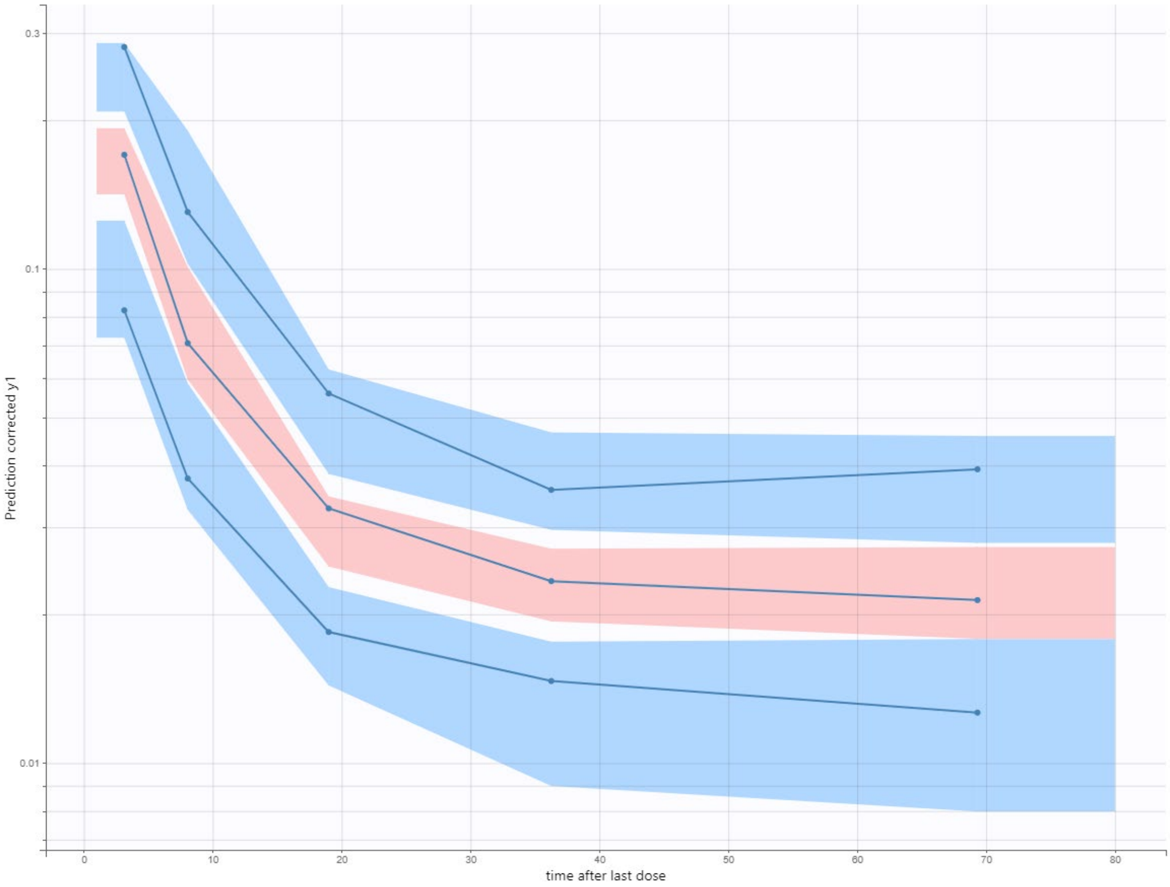

Figure S1_4.2.4 Observed versus predicted concentrations of R-Norketamine.

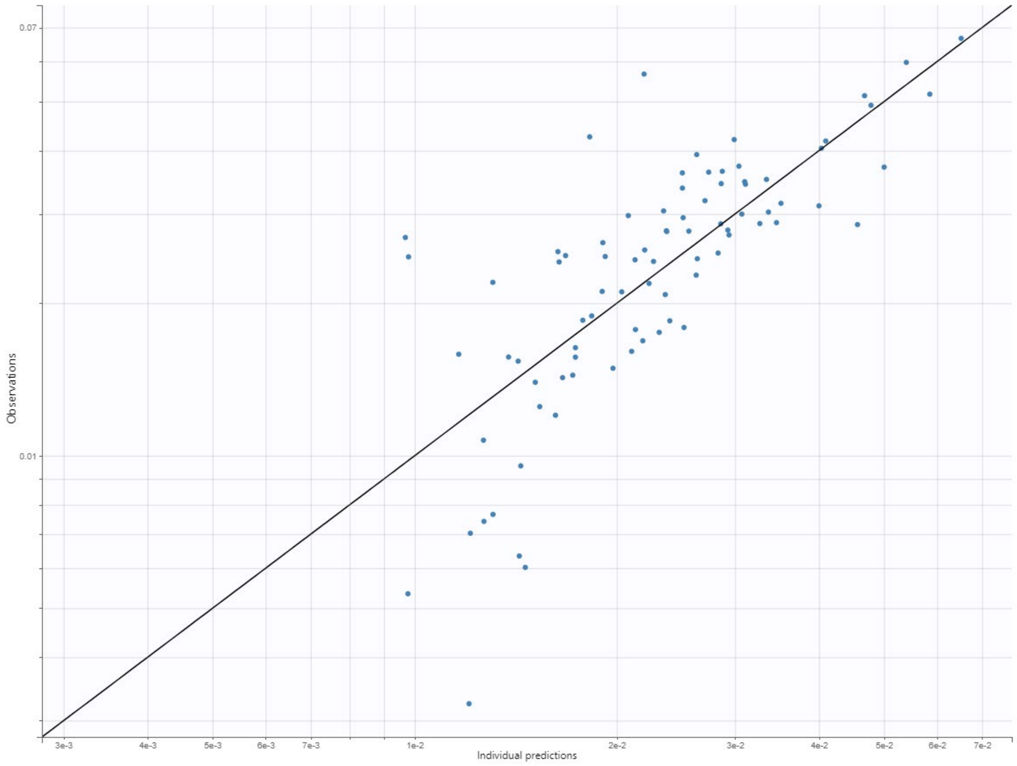

Figure S1_4.2.5 Residual distribution for R-Norketamine

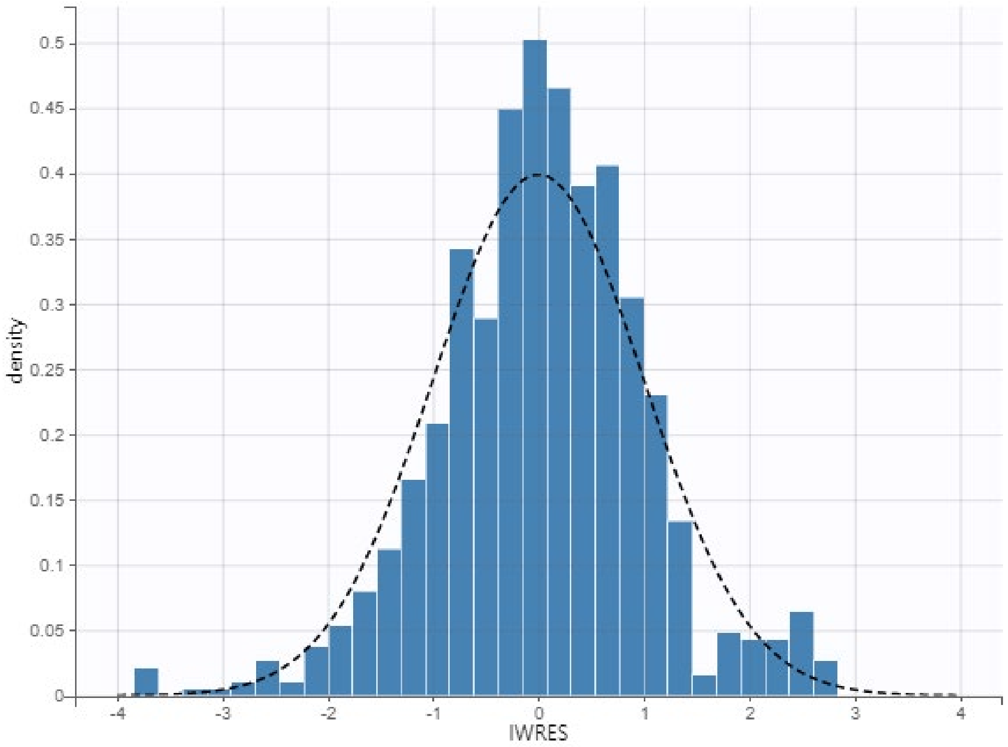

Figure S1_4.2.6 Visual predictive check for R-Norketamine.

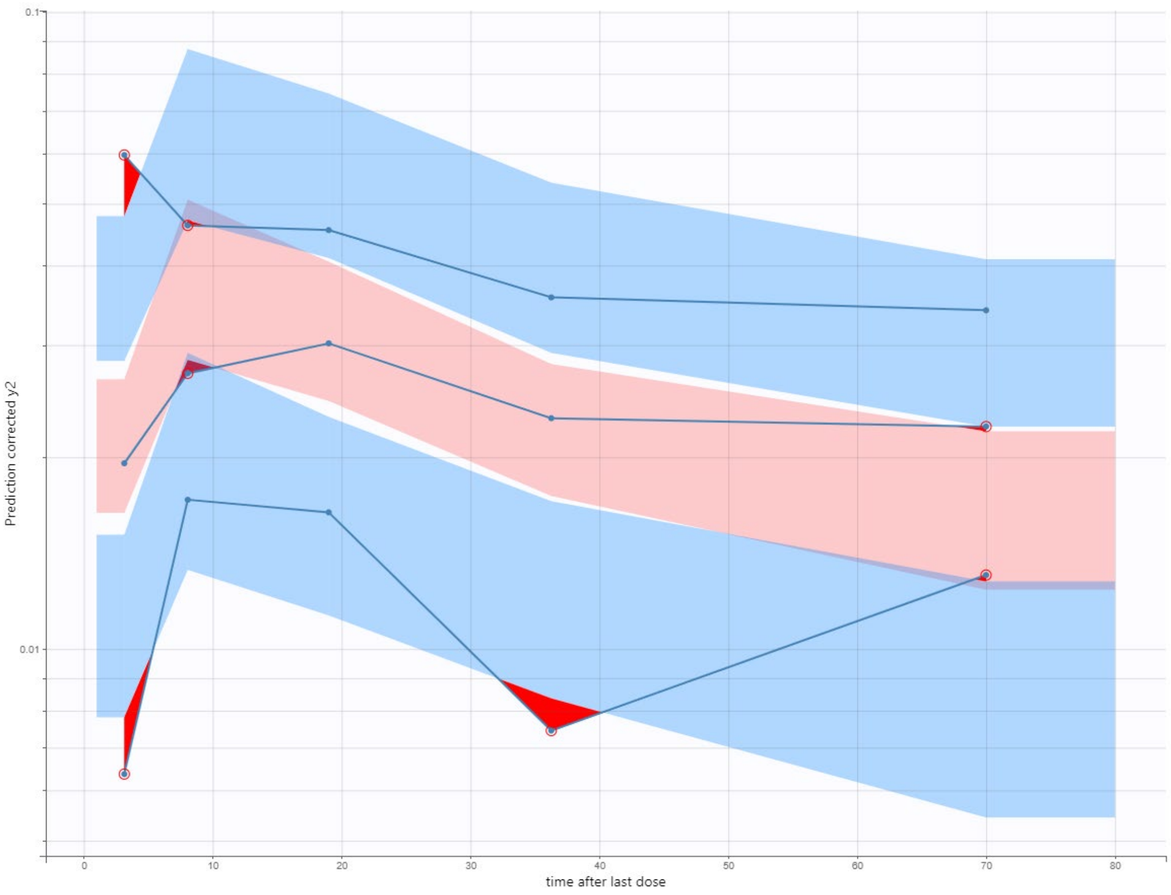

5. Prediction S-ketamine:

Based on the final Population Parent-metabolite model obtained for S-ketamine, a prediction is performed with administration of 0.5 mg/kg IV racemic ketamine over 1 minute, followed by 2 mg/kg/h for 60 minutes, reduced to 1.8 mg/kg/h for further 60 minutes.

Figure S1_5.1 Prediction for S-Ketamine plasma concentration over time (minute) showing median and 5-95% confidence interval divided in 6 areas (5-20%, 20-35%, 35-50%, 50-65%, 65-80%, 80-95%).

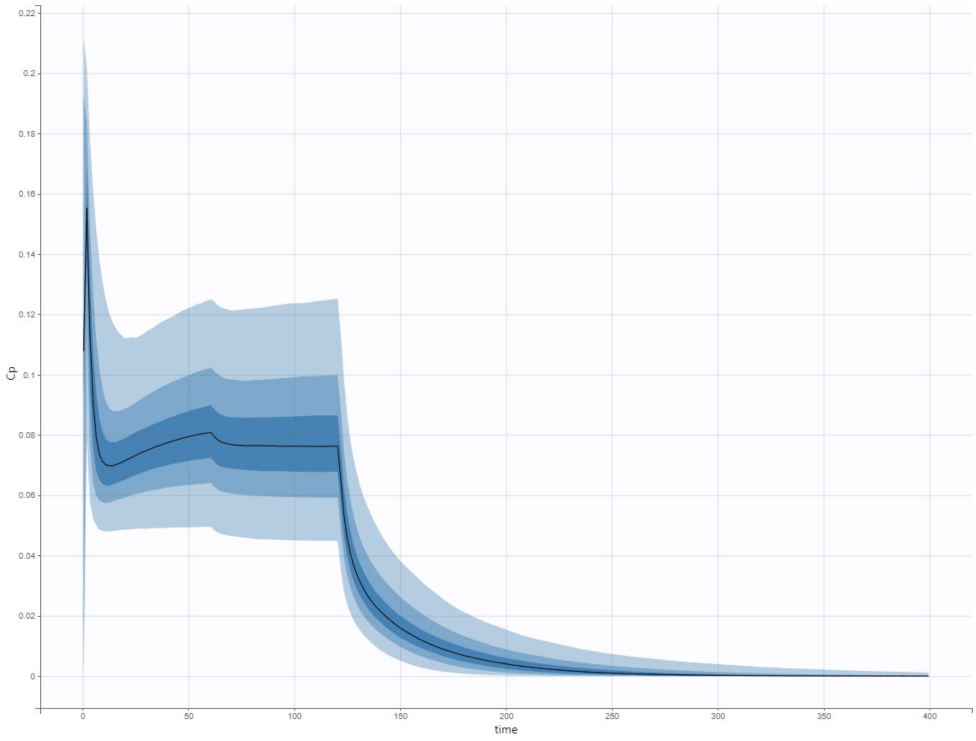

Figure S1_5.1 Prediction for S-Norketamine plasma concentration over time showing median and 5-95% confidence interval divided in 6 areas (5-20%, 20-35%, 35-50%, 50-65%, 65-80%, 80-95%).

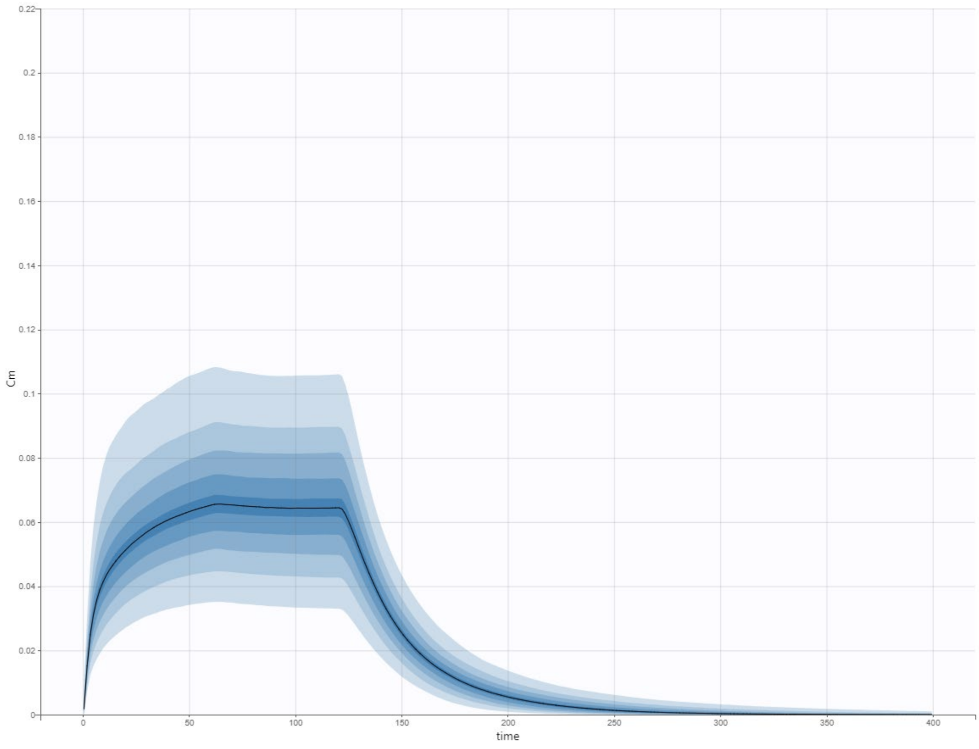

Supplement: Supplementary file 1 [file animals-14-01012-s001.zip › Supplementary material S1.pdf]
